# Long-term molecular surveillance provides clues on a cattle origin for *Mycobacterium bovis* in Portugal

**DOI:** 10.1038/s41598-020-77713-8

**Published:** 2020-11-30

**Authors:** Ana C. Reis, Rogério Tenreiro, Teresa Albuquerque, Ana Botelho, Mónica V. Cunha

**Affiliations:** 1 Centre for Ecology, Evolution and Environmental Changes (cE3c), Faculdade de Ciências da Universidade de Lisboa, Campo Grande, 1749-016 Lisboa, Portugal; 2 Biosystems & Integrative Sciences Institute (BioISI), Faculdade de Ciências da Universidade de Lisboa, Lisboa, Portugal; 3 National Institute for Agrarian and Veterinary Research (INIAV IP), Oeiras, Portugal

**Keywords:** Evolution, Microbiology, Pathogens, Epidemiology

## Abstract

Animal tuberculosis (TB), caused by *Mycobacterium bovis*, is maintained in Portugal in a multi-host system, with cattle, red deer and wild boar, playing a central role. However, the ecological processes driving transmission are not understood. The main aim of this study was thus to contribute to the reconstruction of the spatiotemporal history of animal TB and to refine knowledge on *M. bovis* population structure in order to inform novel intervention strategies. A collection of 948 *M. bovis* isolates obtained during long-term surveillance (2002–2016, 15 years) of cattle (*n* = 384), red deer (*n* = 303) and wild boar (*n* = 261), from the main TB hotspot areas, was characterized by spoligotyping and 8 to 12-*loci* MIRU-VNTR. Spoligotyping identified 64 profiles and MIRU-VNTR distinguished 2 to 36 subtypes within each spoligotype, enabling differentiation of mixed or clonal populations. Common genotypic profiles within and among livestock and wildlife in the same spatiotemporal context highlighted epidemiological links across hosts and regions, as for example the SB0119-M205 genotype shared by cattle in Beja district or SB0121-M34 shared by the three hosts in Castelo Branco and Beja districts. These genomic data, together with metadata, were integrated in a Bayesian inference framework, identifying five ancestral *M. bovis* populations. The phylogeographic segregation of *M. bovis* in specific areas of Portugal where the disease persists locally is postulated. Concurrently, robust statistics indicates an association of the most probable ancient population with cattle and Beja, providing a clue on the origin of animal TB epidemics. This relationship was further confirmed through a multinomial probability model that assessed the influence of host species on spatiotemporal clustering. Two significant clusters were identified, one that persisted between 2004 and 2010, in Beja district, with Barrancos county at the centre, overlapping the central TB core area of the Iberian Peninsula, and highlighting a significant higher risk associated to cattle. The second cluster was predominant in the 2012–2016 period, holding the county Rosmaninhal at the centre, in Castelo Branco district, for which wild boar contributed the most in relative risk. These results provide novel quantitative insights beyond empirical perceptions, that may inform adaptive TB control choices in different regions.

## Introduction

The control and eradication of infectious diseases can be very difficult for pathogens that are able to thrive across multiple host species. This is the case of animal tuberculosis (TB), an infectious disease mainly caused by *Mycobacterium bovis* (*M. bovis*), which circulates within livestock and across several wildlife species worldwide, through direct and indirect routes^1–4^. Animal TB affects livestock productivity and trading, animal welfare, and also biodiversity, as it globally or locally threatens ecosystem services. The zoonotic potential of *M. bovis* bacteria is also liable as new infection human cases are reported every year^5,6^.

Cattle (*Bos taurus*) is described as the main host species for *M. bovis*, while several wildlife hosts can support pathogen maintenance, depending on the ecosystem and epidemiological scenario. Over the years several molecular reports demonstrated inter-species transmission between cattle and wildlife, and spatial interaction studies associated wildlife as a risk factor for TB infection in cattle. Badger (*Meles meles*) in United Kingdom and Republic of Ireland; African buffalo (*Syncerus caffer*) in South Africa, brushtail possums (*Trichosurus vulpecula*) in New Zealand, white-tailed deer (*Odocoileus virginianus*) in EUA, and red deer (*Cervus elaphus*) and wild boar (*Sus scrofa*) in the Iberian Peninsula (Portugal and Spain) are the best characterized wildlife reservoirs^5,7–10^. In Iberian ecosystems, cattle and non-bovine livestock species managed in extensive husbandry, and red deer, wild boar, fallow deer and small carnivores compose the host community that can support *M. bovis* maintenance^11^. Some spatial clusters of infection in central and south-eastern regions of Portugal, in the periphery of the Iberian TB core area, have been detected, where average TB prevalence may reach as much as 50% in wild ungulate populations alone^7,10,12^. Expansion from the core Iberian area is believed to be driven by high host densities, artificial management and super-shedder wild populations^13,14^.

**Figure 1:**
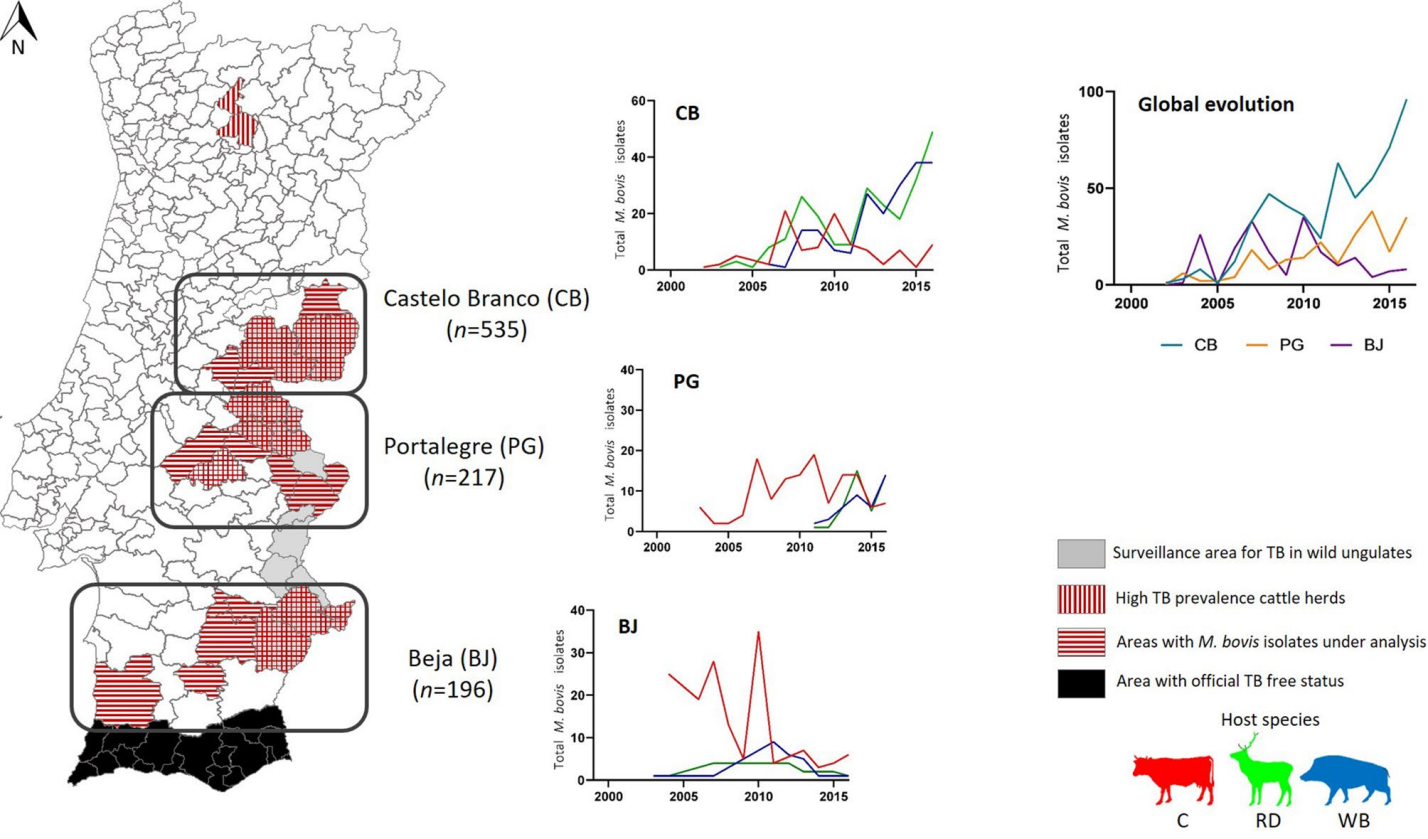
Map of mainland Portugal highlighting the high TB prevalence area for cattle and the epidemiological risk area for wild ungulates (red deer and wild boar) and evidencing the geographic distribution of *M. bovis* isolates under analysis. The middle plots represent the evolution of the total number of *M. bovis* isolated from the different host species (red for cattle (C), green for red deer (RD) and blue for wild boar (WB)) sampled per geographic region, and the plot at the right compares the global number of *M. bovis* isolates per geographic region.

In Portugal, a national eradication program for animal TB in cattle population commenced in 1987, being presently based on the detection and culling of reactors to the single intradermal comparative tuberculin (SICCT) test and the interferon-γ test of cattle aged over 6 weeks, also including routine surveillance at slaughterhouses, mandatory sanitary classification of herds and regions, compulsory slaughter of positive animals, compensation to owners of slaughtered animals and pre-movement assessments^15^. Bacteriological culture and histopathology are the officially accepted diagnostics to confirm animal TB infection. Portugal has made significant progress in the control of bovine TB since the nationwide implementation of the eradication program approved by the European Union in 1992 (Council Decision 92/299/CE). Currently, the program covers mainland Portugal and the autonomous regions of Azores and Madeira^16^. During the last decade, TB herd prevalence remained low, varying between 0.11% in 2008 and 0.29% in 2017, with a peak in 2010 (0.91%)^17^. However, the national epidemiological indicators reflect different contexts throughout mainland Portugal, with TB herd prevalence ranging from 0.08% in the North up to 1.24% in Alentejo (south) in 2017^17^. Algarve, in the extreme south of Portugal, is the single region that already achieved the officially TB-free status (Decision 2012/204/EU), where a specific surveillance program applies for the maintenance of this status (Fig. 1). The eradication program has suffered changes along the years since its first implementation. While in vivo surveillance and SICCT testing used to be homogenous across cattle herds, it has recently adapted towards the evolution of the epidemiological situation. There are now regional differences in the application of the surveillance scheme, according to herd type (beef, dairy, breeding), TB history and geographical location.

The heterogeneity of TB herd prevalence across mainland Portugal indicates that some regional circumstances may favour intraspecific and interspecific *M. bovis* transmission, such as: (1) the management in extensive husbandry of livestock in the central and south regions that promote aggregation and enable the direct and indirect interaction of domestic with wild animals in communal pastures; (2) the wide host community of wildlife species potentially susceptible to TB that free-range in Portugal; and (3) the overabundance of widely distributed big game species that are artificially managed for hunting purposes, namely red deer and wild boar.

Following several scientific publications reporting *M. bovis* occurrence in wild populations across two regional foci^10,18–20^ and the field knowledge reported by veterinary services of some municipalities, combined with evidence for growing abundances of wild ungulates and the proliferation of big game hunting, in 2011 the national authorities defined an epidemiological risk area for big game species in south-central Portugal, along the border with Spain, where specific surveillance measures apply^21^. All big game specimens hunted in this risk area are mandatorily subjected to *post-mortem* examination in the field by a credentialed veterinarian. Samples from suspected TB lesions are collected and forwarded to the National Reference Laboratory (NRL) to confirm TB. Hunting by-products and animals with TB-compatible lesions (fully rejected) are disposed according to European Regulations (EC) no. 1069/2009 and 854/2004^21^.

The resilience of *M. bovis* in wildlife hosts remains a challenge for TB eradication in the cattle population and raises cattle and wildlife management issues in addition to public health concerns. The national context in Portugal is of special complexity, since *M. bovis* is maintained in this multi-host system with at least three maintenance hosts. Their single or joint contribution for *M. bovis* transmission and spatial patterns are not yet quantified. Although reports of *M. bovis* occurrence in other mammal species (e.g. caprine, ovine, carnivores) have remained occasional^22,23^, the role of each species in these transmission processes is unknown for now.

Under these complex circumstances, appropriate epidemiological studies applied to high burden areas are required. So, in this work, we have focused on perceived hotspot areas where the livestock-wildlife interface is prominent, with widely distributed cattle herds of varying dimensions and abundant wild ungulate populations. We take advantage of a large collection of *M. bovis* isolates which has been gathered for almost two decades in the NRL and that can be exploited at the molecular level. A detailed molecular approach making use of spoligotyping (Spacer oligonucleotide typing) and MIRU-VNTR (*Mycobacterial Interspersed Repetitive Units-Variable Number Tandem Repeats*)^22–27^, combined with Bayesian clustering inference analyses, phylogenetic and network analyses, was envisaged with the aim to refine knowledge on the *M. bovis* population structure and to assess the spatiotemporal distribution of the most common genotypes, enabling the inference of the spatial risk, the most epidemiologically relevant hosts and the most probable transmission routes.

## Methods

**Figure 2:**
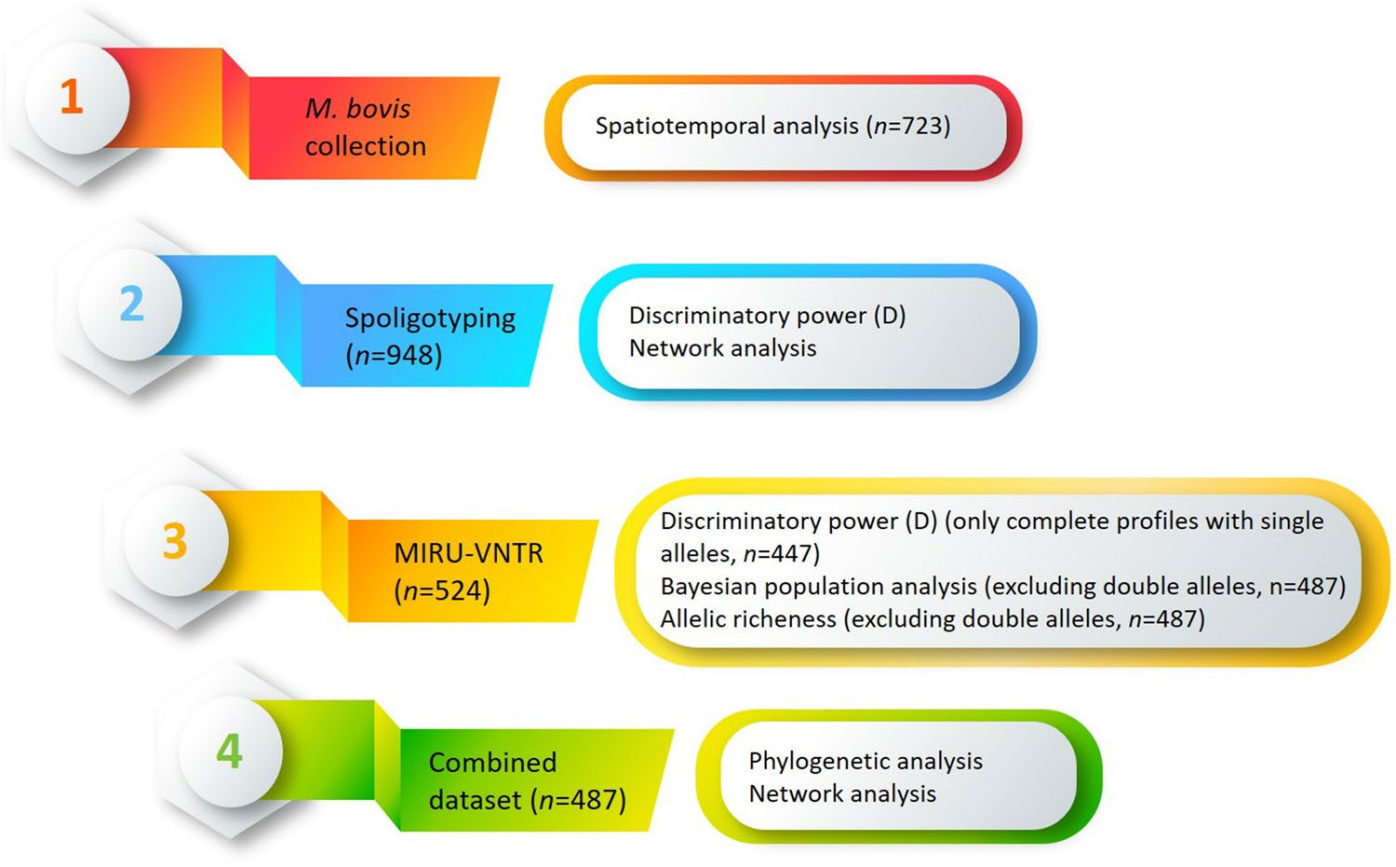
General workflow followed in this study (general template obtained in https://tinyppt.com/).

The general workflow followed in this study that involved genotypic characterization by two methods and statistical analyses is described in detail in Fig. 2.

### Study area

The study area comprises the three regions regarded in Portugal as the main hotspot areas for animal TB at the livestock-cattle interface. They are enclosed in the districts (administrative level sample unit) of Castelo Branco, Portalegre and Beja, which locate on inner central and south Portugal, within the epidemiological risk area for TB in big game species that simultaneously holds chronically infected cattle herds that are managed in an extensive regimen (Fig. 1). Castelo Branco was the most represented district, with 535 *M. bovis* isolates, followed by Portalegre, with 217, and Beja, with 196 isolates (Fig. 1). The study area is simultaneously used for livestock production and big game hunting activities.

### Bacterial isolation and identification

Nine hundred and forty-eight *M. bovis* isolates were recovered (*n* = 948), in a 15-year period (2002–2016), from three host species, namely cattle (*Bos taurus*) (*n* = 384), red deer (*Cervus elaphus*) (*n* = 303) and wild boar (*Sus scrofa*) (*n* = 261) (Fig. 1).

This 15-year molecular analysis concerns three time periods: period 1 (2002–2008; 7 years) in which herd prevalence steadily decreased and surveillance in wildlife was sporadic and uneven; period 2 (2009 and 2012; 4 years) in which national herd prevalence raised more than fourfold and carcass examination of hunted big game species became mandatory in the epidemiological risk area from 2011 onwards; and, finally, period 3 (2013–2016; 4 years) that was characterized by a decrease in herd prevalence after mandatory augmented surveillance by stakeholders, both at the livestock and wildlife flanks.

Sample collection was performed within an official context according to specific national legislation or in the context of research projects held by the NRL. No animals were slaughtered or hunted for this study purpose. Cattle samples (lungs, lymph nodes, liver and/or spleen) were obtained after sanitary slaughter following previous positive intradermal testing or from evidence of macroscopic TB-like lesions at routine abattoir inspection. Wildlife samples (lungs, lymph nodes, liver and/or spleen) were collected from hunted animals presenting TB-compatible lesions that were detected during veterinarian examination in the field, which is obligatory for 19 counties (municipal level) enclosed in the hotspot areas.

Animal tissue samples were pooled and processed in a biosecurity level 3 facility at INIAV, IP (National Institute for Agrarian and Veterinary Research), the NRL for animal TB diagnosis, following the protocol guidelines recommended in the OIE Manual for Terrestrial Animals (a detailed procedure is described in Cunha et al*.*^7^); and inoculated onto Stonebrink (Biogerm, Portugal) and Löwenstein-Jensen pyruvate (Biogerm, Portugal) solid media and liquid medium (BACTEC 9000 MB system, Becton Dickinson Microbiology Systems). Cultures were incubated at 37 °C and inspected weekly for growth for a minimum period of 12 weeks.

Pelleted cultures were heat-killed to extract the DNA and species identification was performed by restriction fragment length polymorphism analysis of PCR-amplified *gyrB* gene (PCR-REA) as described in Cunha et al*.*^7^ or using commercially available systems [GenoType Mycobacterium (Hain diagnostics, Germany)], according to manufacturer instructions. In all PCR batch assays, DNA from two positive controls (*M. tuberculosis* H37RV and *M. bovis* BCG) and a no-template control (purified water) were included.

### Spatiotemporal cluster analyses

A space–time analysis with a multinomial probability model^28^ was performed in SaTScan (version 9.6, https://www.satscan.org/) to assess the influence of host species on spatio-temporal risk clustering. In total, 249 TB cases in cattle, grouped in 135 livestock herds, and 474 TB cases in wildlife hosts, grouped in 91 officially-delimited hunting areas, were considered, meaning 76.4% of the global dataset, for which geographic coordinates of the location centroids of herds/hunting areas, were available. A database was created by geocoding each TB case to the geographic coordinates of the corresponding livestock herd or officially-delimited hunting area and no substantial change in geographic coordinates was assumed over the study period. The multinomial probability model applied here evaluates whether there are any clusters where the distribution of TB cases is different from the rest of the region under study. To operationalize the model, the study area is scanned using cylinders of varying sizes, with the base representing space and the height representing time, and the number of cases inside the cylinders is compared to the case expectations outside. Space–time clusters were identified at a maximum temporal and spatial window of 50.0% of the study period and case data, respectively. It was only allowed one set of geographical coordinates per location (herd/hunting area) and no overlapping clusters were permitted. Statistically significant clusters were evaluated at a *p*-value < 0.05, using Monte Carlo permutations with 999 replications of the dataset, under the null hypothesis of complete spatial randomness.

The map was generated using QGIS (Quantum GIS development Team 2018, version 3.10, https://www.qgis.org/en/site/) to visualize spatial‐temporal clusters and outbreak locations.

### Molecular characterization of *M. bovis* isolates

Spoligotyping was performed with in-house made membranes following the standard published protocol^29^, with the modifications described by Botelho et al*.*^30^. The presence or absence of the 43 spacers contained in the Direct Repeat (DR) *locus* was represented by a binary code. Spoligotyping patterns (prefix SB followed by four digits) were obtained from the Spoligotype Database website (http://www.mbovis.org/index.php). DNA from *M. bovis* BCG and *M. tuberculosis* H37Rv isolates were used as positive controls and purified water as no-template control.

The relative proportion of each one of the top six most common spoligotyping profiles, over the remaining in the different study years was obtained. A global and regional trend analysis was performed by applying a linear regression.

A representative collection of isolates (*n* = 524) was further subjected to MIRU-VNTR analyses. Two selection criteria were applied: firstly, isolates were selected to cover all spoligotyping groups; and secondly, to cover in equal proportion different host species, geographic regions and year of isolation. Moreover, nine of the 29 orphan spoligotypes (i.e. with only one isolate) were also included.

MIRU-VNTR typing was performed using a previously established set of 8-*loci* panel constituted by VNTR3232, ETR-A, ETR-B, ETR-C, QUB11a, QUB11b, MIRU4 and MIRU26^20^. These *loci* were amplified by PCR, in single reaction tubes, using the primers and cycling conditions previously described by Duarte et al*.*^20^. Positive control (DNA extracted from *M. bovis* BCG) and a no-template control (purified water) were included in all PCR assays. PCR products were separated in 2% agarose gels, prepared in Tris Buffer EDTA 1×, at 90 V for 3 h. The number of tandem repeats in each amplified *locus* was attributed by correlation with the amplicon size according to previously published tables^31^.

Complete profiles were grouped by similarity in MIRU-VNTR types (M types). Profiles with non-amplifiable *loci* were excluded from this classification.

A small subset of the MIRU-VNTR results (< 10%) reported in this study have been previously published by Duarte et al*.*^20^ (*n* = 23, between 2002 and 2007) and by Cunha et al*.*^7^ (*n* = 27, between 2007 and 2009), but were included to span the analyses for a wider period.

A case was considered as polyclonal infection when two amplification products for one or more MIRU-VNTR *loci* were detected, as in Reis et al*.*^32^. In these cases, the presence of double alleles was confirmed by repeating the PCR and electrophoresis experiments. For the subgroup with double alleles (*n* = 37), four additional *loci* (MIRU16, MIRU40, ETR-E and QUB26) were added to the initial panel with the purpose of further discriminating the isolates, following the rational indicated by Reis et al*.*^32^. Similar to the 8-*loci* panel, these four additional *loci* were amplified by PCR, in single reaction tubes, using the primers described by Supply et al*.*^33^ for MIRU16, MIRU40 and ETR-E, and Skuce et al*.*^34^ for QUB26. The cycling and electrophoresis conditions applied were equal to the ones already described for the previous panel.

The genotypic profiles were defined by the combination of spoligotyping profile and MIRU type.

The calculation of the discriminatory power (D), that is the average probability that the typing method will assign a different type to two unrelated isolates sampled in the population, for both typing methods, and the individual allelic diversity (h) of the *loci* panel tested were obtained. These parameters were calculated based on Simpson's index of diversity, as described by Hunter and Gaston^35^, using an online tool available on the website *In silico simulation of molecular biology experiments* (In silico, 2018)^36^. Only complete profiles with single alleles were considered for the calculation of the discriminatory power of MIRU-VNTR typing.

### Network analyses

To explore the relationships established within each host-geographic region pair, two networks involving spoligotyping (*n* = 948) or genotypic profiles (*n* = 487) (i.e. based on spoligotyping and MIRU) were visualized with Gephi (version 0.9.2, https://gephi.org/). Each host-geographic region combination was considered a node, and the number of shared spoligotyping or genotypic profiles were used as connection lines between nodes. The connection lines were established based on absolute values; no statistical transformation was performed.

### Phylogenetic analysis

BioNumerics software (version 6.6, http://www.applied-maths.com/bionumerics) (Applied Maths, Sint-Martens-Latem, Belgium) was used to visualize evolutionary relationships among the *M. bovis* isolates by generating Minimum Spanning Trees (MSTs), using a combined dataset composed by spoligotyping and 8-*loci* MIRU-VNTR (*n* = 487). Profiles with double alleles were excluded from these analyses.

A composite character experiment was created with spoligotyping and MIRU-VNTR data, and an advanced cluster analysis was performed using the categorical option as a similarity coefficient. The single *locus* variant was used as a criterion for maximum distance between nodes.

### Bayesian population analysis

To further explore the population structure of *M. bovis* isolates, a Bayesian clustering algorithm, implemented in the software STRUCTURE (version 2.3.4, https://web.stanford.edu/group/pritchardlab/structure.html)^37^ was applied to a dataset composed by MIRU-VNTR profiles (*n* = 487). Bayesian inference method and MST analysis were applied to the same *M. bovis* isolates. STRUCTURE was used to infer the most likely number of putative ancestral populations (K), and to assign individuals to these populations. It was ran using the admixture model as an ancestry prior model. The admixture model considers that the data may have been originated from the admixture of K putative ancestral populations at some time in the past, so it was selected because it is a more flexible model and, thus, suitable for dealing with the complexities of a real data population. Five independent simulations were performed using Markov Chain Monte Carlo (MCMC) with an initial 10.000 ‘burn-in’ period and 100.000 iterations, following ‘burn-in’. For each simulation, K values were tested between 2 and 15, with 10 replicated runs for each value of K.

To estimate the number of putative ancestral populations, delta K was calculated by the Evanno method^38^, using STRUCTURE HARVESTER^39^. The estimate of delta K is based on the rate of change in the log probability of data between successive K values.

The individual Q matrix corresponds to the estimated ancestry coefficients for each individual, that is the part of the genome that each individual inherited from ancestors. A threshold of Q ≥ 0.50 was applied to assign individuals to ancestral populations.

New MST analyses were then performed using BioNumerics software (version 6.6), according to the criterion described in “Phylogenetic analysis” section, and identifying *M. bovis* by the classification provided by STRUCTURE analysis.

### Allelic richness

For allelic richness analyses, *M. bovis* isolates were grouped according to geographic region and populations defined by STRUCTURE. The mean allelic richness of each *M. bovis* group was evaluated using the statistical method of rarefaction implemented in the software HP-RARE (version 1.0, https://www.montana.edu/kalinowski/software/hp-rare.html)^40^, which standardizes samples of different sizes, compensating for sampling disparity, so that the allelic diversity across large and small sample datasets could be compared.

### Statistical analyses

The statistical significance between the associations of spoligotyping profiles proportion and geographic region or host species were tested using a chi-square test (α = 0.05). An unbiased estimate of the exact significance level was performed through Monte Carlo approximation with confidence interval of 99% and 10,000 repeated sampling. Chi-square test (α = 0.05) was also used to evaluate the statistical significance of the association between *M. bovis* ancestral populations defined by STRUCTURE software and geographic region or host species. Contingency tables and tests were performed in IBM SPSS Statistics (version 24.0.0, https://www.ibm.com/analytics/spss-statistics-software). Results were considered to be significant if *p* < 0.05.

## Results

### Demography analyses of *M. bovis* isolates

The *M. bovis* dataset considered in this study is well represented as far as time, space and animal hosts are concerned, however the strain set of the first period, especially from 2002 to 2006, is restricted (*n* = 85), corresponding to only 9% of the total dataset, which may be explained by reduced and uneven surveillance during that time period. The number of isolates has two peaks in 2007 and 2010 and values rise since 2013, coinciding with the increase in TB surveillance of the wildlife component.

Until 2006, Beja was the most represented region (cumulative number of isolates = 46), but since then and until 2016, Castelo Branco (*n* = 511) outnumbered the number of *M. bovis* isolates recovered (*n* = 150 for Beja and *n* = 202 for Portalegre) (Fig. 1).

Cattle was the most represented host species until 2011 (*n* = 258), then wildlife species were sampled in higher numbers (*n* = 437) and with similar proportions each. *M. bovis* became regularly isolated from wildlife hosts after 2011, which is related with the settlement of the epidemiological risk area for TB in game species (Fig. 1).

**Figure 3:**
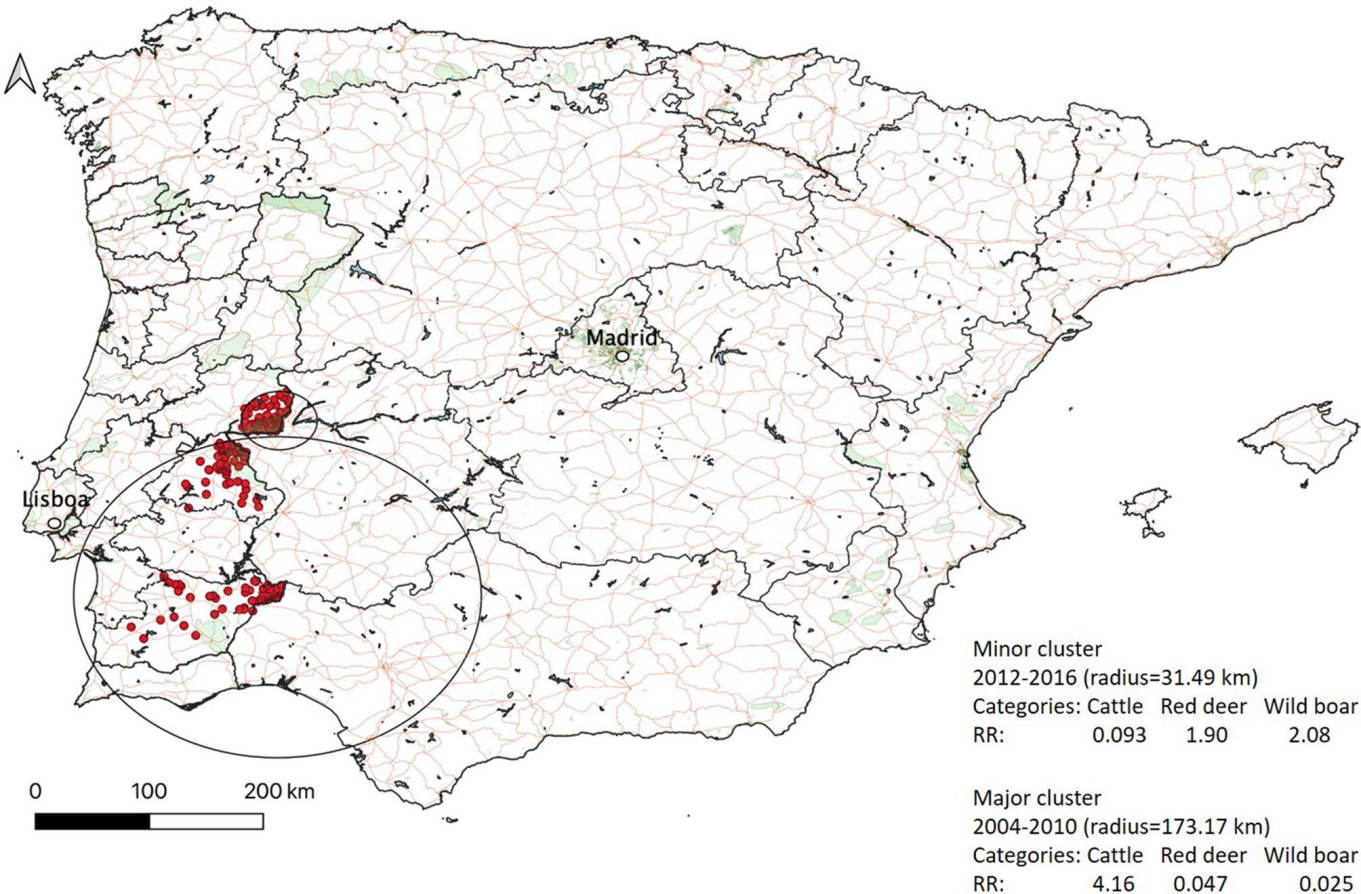
Multinomial space–time clusters (black-lined circles) of *M. bovis* calculated for the 2002–2016 period. Categories refer to host species. Relative Risk (RR) estimates indicate whether or not, greater than expected numbers occurred in a given category. RR > 1 represents a greater than expected number of individuals of a certain category inside the spatial-time cluster compared to outside. The radius of each space–time cluster is indicated. Map was generated with QGIS software (version 3.10, https://www.qgis.org/en/site/).

The space–time clustering behaviour of TB cases was evaluated with a multinomial probability model, in a total of 723 cases, reported between 2002 and 2016, in cattle (*n* = 249), red deer (*n* = 247) and wild boar (*n* = 227). Two significant clusters were identified (*p*-value = 0.001) and were both composed by TB cases originated from the three host species (Fig. 3). The 173.17 km-radius spatial cluster in Barrancos county, Beja district, included a total of 108 TB cases (*n* = 105 for cattle, *n* = 2 for red deer and *n* = 1 for wild boar) and persisted between 2004 and 2010, pointing out to a remarkable higher relative risk (RR) for cattle (RR = 4.16), when comparing with wild boar (RR = 0.025) and red deer (RR = 0.047) (Fig. 3). The other cluster, with 31.49 km-radius, holds the county Rosmaninhal at the centre, in Castelo Branco district, and embraces 270 TB cases (*n* = 13 for cattle, *n* = 131 for red deer and *n* = 126 for wild boar) from 2012 to 2016. In this cluster, wild boar (RR = 2.08) presents a higher relative risk compared to red deer (RR = 1.90) and cattle (RR = 0.093) (Fig. 3).

### Genotypic diversity of *M. bovis*

Spoligotyping was used as first-line genotyping tool for the selected 948 *M. bovis* isolates, distinguishing 64 patterns with varying prevalence (overall discriminatory power, D = 0.92) (Supplementary Fig. S1). Three spoligotypes, SB2529, SB2530 and SB2531, were recorded for the first time, and were introduced at the *M.bovis*.org database. Twenty-nine patterns with lower frequency (encompassing 2 to 42 isolates) and 29 orphan patterns represented 32% and 3% of total isolates, respectively (Supplementary Fig. S1 and Supplementary Table S1).

**Figure 4:**
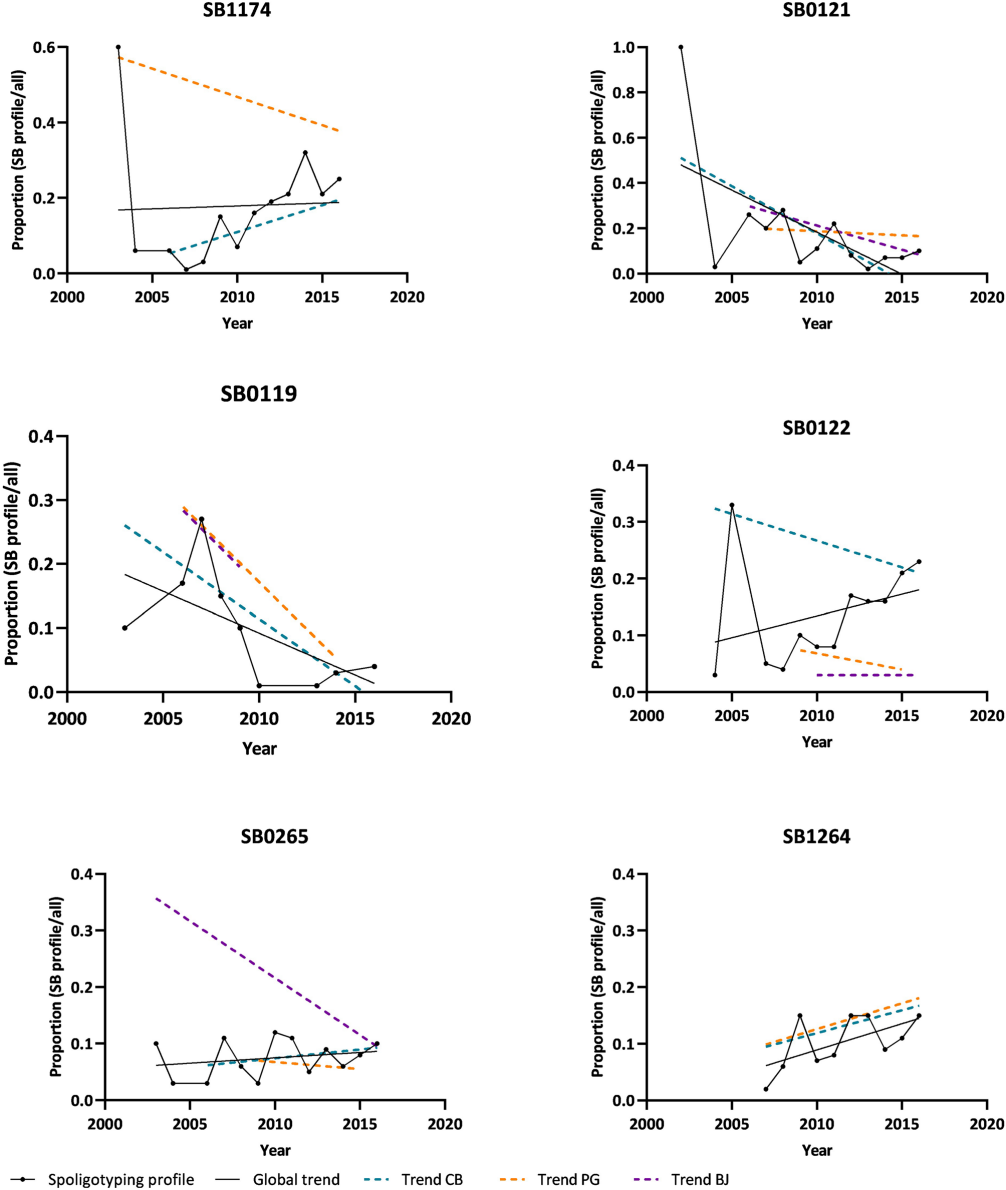
Variation in the proportion of *M. bovis* isolates included within the top 6 spoligotyping profiles. The solid line represents global predictions from a linear regression with 95% confidence interval, while punctuated lines represent the prediction for each geographic hotspot. Each graph is identified by the spoligotyping profile.

SB1174 (*n* = 158; 16.7%), SB0122 (*n* = 123; 13.0%), SB0121 (*n* = 111; 11.7%), SB1264 (*n* = 92; 9.7%), SB0265 (*n* = 75; 7.9%) and SB0119 (*n* = 57; 6.0%) were the top 6 profiles and together account for 65% of total isolates (Supplementary Fig. S1, Table S1). These six profiles were documented in the three host species and, with the exception of SB1174 and SB1264, they were distributed across the three geographic regions under analysis. The proportion of SB0119 and SB0121 varied across the study period, exhibiting a global negative trend, while SB0122 and SB1264 showed a positive tendency (Fig. 4).

For a subset of 524 *M. bovis* isolates (selected based on host species, location, year—see “Methods” section), a panel of 8-*loci* MIRU-VNTR was applied, resulting in 447 complete profiles with single alleles, grouped by similarity in 157 MIRU types and following the denomination of Cunha et al.^7^; 37 complete profiles with double alleles in one or more *loci*; and 40 profiles with one systematically un-amplified *locus* (Supplementary Fig. S2, Table S2).

The majority of MIRU type groups (*n* = 95; 60.1%) were singletons, whereas a minority comprised 2 to 37 isolates (Supplementary Fig. S2). The six most common MIRU types (M34, M205, M219, M225, M251 and M261) account for 35% of sub-typified isolates (Supplementary Table S2). The contribution of singletons for global analysis was higher in the first time period, representing 26% of total MIRU types, whereas in the last considered time period singletons decreased to 21%.

The largest MIRU type groups (M261, M34 and M225), encompassing over 30 isolates each, were found in the three host species under analysis; whereas M34 was recorded in the three regions, and M225 and M261 were restricted to Castelo Branco and Portalegre (Supplementary Table S2).

The overall discriminatory power of MIRU-VNTR typing was higher than spoligotyping (D = 0.97) and the allelic diversity of individual *locus* ranged from 0.28 to 0.68, in decreasing order: ETR-A (*h* = 0.68), QUB11b (*h* = 0.58), ETR-B (*h* = 0.57), ETR-C (*h* = 0.53), VNTR 3232 (*h* = 0.42), QUB11a (*h* = 0.41), MIRU4 (*h* = 0.32) and MIRU26 (*h* = 0.28). All MIRU-VNTR *loci* were polymorphic, ranging between three (MIRU4) and 10 (VNTR3232) different alleles *per locus*.

MIRU-VNTR typing enabled a finer discrimination of *M. bovis* isolates within the same spoligotype group, with the exception of two isolates with profile SB1273 that presented the same MIRU type. Therefore, the combined dataset of results from both typing methods enabled the definition of 208 genotypic profiles (Supplementary Table S3). There were genotypic profiles common to the three host species, but never to the three geographic regions under analysis; however, common profiles across bordering regions (Castelo Branco and Portalegre or Portalegre and Beja) were found.

Polyclonal infection was suggested in 37 cases, through the identification of double alleles in one to four *loci*. Double alleles were registered across all *loci*, with the exception of QUB11a, and in the majority of profiles (*n* = 31; 83.8%) were restricted to one single *locus* (Supplementary Table S4). *M. bovis* genotypic variants were isolated from the three host species and geographic regions throughout the 2006–2015 period. In a small proportion of cases (*n* = 6), restricted to wild hosts from Castelo Branco, double alleles were recorded in two to four *loci* (Supplementary Table S4).

### *M. bovis* genotypic diversity across TB hotspot regions

**Figure 5:**
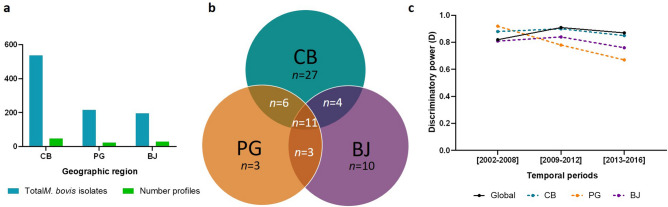
Spoligotyping analysis per geographic region: (**a**) comparison between the total number of *M. bovis* isolates and the spoligotyping profiles registered; (**b**) common and specific-spoligotyping profiles; (**c**) evolution of the discriminatory power (D values) of spoligotyping for the global dataset (black line) and for each geographic region. Blue represents Castelo Branco (CB), orange Portalegre (PG) and purple Beja (BJ).

The most represented region, Castelo Branco, was also the region that yielded the highest number of spoligotypes, with the identification of 48 profiles (D = 0.8974), followed by Beja, with 28 (D = 0.8966), and Portalegre, with 23 (D = 0.7762) (Fig. 5). Analysis of spoligotyping diversity evolution through time, reveals an overall reduction in the three TB hotspots (Fig. 5). From time period 1 to time period 3, D values varied between 0.88 to 0.85 in Castelo Branco, 0.92 to 0.67 in Portalegre and 0.81 to 0.76 in Beja.

Eleven spoligotypes are common to the three geographic regions, accounting for more than half of *M. bovis* isolates. The panel of Castelo Branco-specific spoligotypes (56%) was higher when comparing with Beja (36%) and Portalegre (13%) (Fig. 5, Supplementary Table S1).

The prevalence rates varied across geographic regions, with SB0122 (21.6%) being most commonly reported in Castelo Branco, SB1174 (43.5%) in Portalegre and SB0265 and SB1190 (15.3%) equally prevalent in Beja (Supplementary Table S1).

In Castelo Branco and Portalegre, the cumulative percentage of the six most common profiles increased by 20%, representing 78 and 81%, respectively, of the dataset in the time period 3. In Beja, profiles SB1174 and SB1264 were substituted by SB0295 and SB1190, and this cumulative evolution was subtle, from 61% in period 1 to 76% in period 3.

Statistically significant associations were established within the spoligotyping profiles and the three locations. Beja was the district with more positive associations established (*p*-value < 0.01), wherein seven profiles were highlighted (SB0119, SB0120, SB0140, SB0265, SB0295, SB1172 and SB1190); Castelo Branco with four (SB0122, SB1195, SB1264 and SB 1266); and, finally, Portalegre with three (SB1167, SB1174 and SB1483) (*p*-value < 0.01) (Supplementary Table S5).

MIRU-VNTR typing grouped the 254 isolates from Castelo Branco into over 100 distinctive MIRU types, while Portalegre and Beja isolates were split into 48 and 35 different MIRU types, respectively (Supplementary Fig. S3). M34 (15.9%) was the most frequent in Beja; M225 (13.4%) in Castelo Branco; and M261 (21.4%) in Portalegre (Supplementary Table S2).

The discriminatory power of MIRU-VNTR was higher than spoligotyping (D = 0.97) and the stratified analysis also disclosed similar values. ETR-A and MIRU26 were the *loci* with highest and lowest allelic diversity, respectively, both in Castelo Branco and Portalegre, while in Beja, QUB11a was the most discriminatory and QUB11b the least.

Three MIRU types (M34, M109 and M199), encompassing 42 isolates, were common to the three geographic regions (Supplementary Fig. S3, Table S2). However, when combining spoligotyping and MIRU type, only adjacent geographic regions presented common genotypic profiles, in a total of 19 common to Castelo Branco and Portalegre and 2 common to Portalegre and Beja (Supplementary Table S3).

### *M. bovis* genotypic diversity across host species

**Figure 6:**
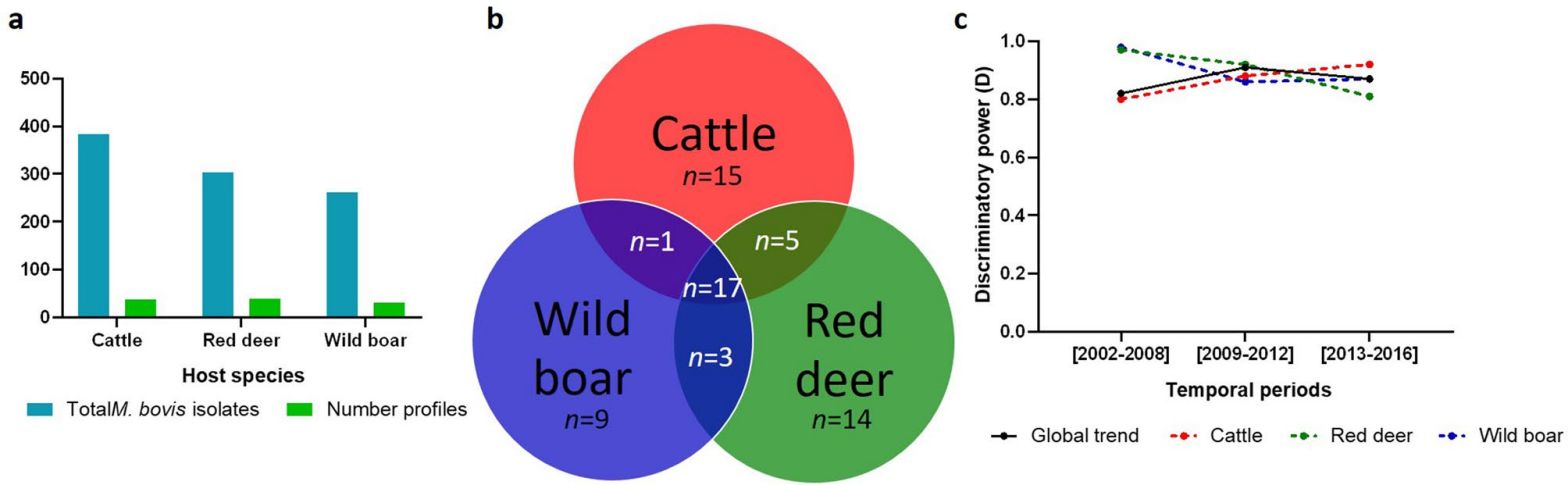
Spoligotyping analysis per host species: (**a**) comparison between the total number of *M. bovis* isolates and the spoligotyping profiles registered; (**b**) common and specific-spoligotyping profiles; (**c**) evolution of the discriminatory power (D values) of spoligotyping, for the global dataset (black line) and for each host species. Red represents cattle (C), green red deer (RD) and blue wild boar (WB).

Red deer yielded the highest number of spoligotyping profiles, with the 303 *M. bovis* isolates being grouped into 39 profiles, when comparing with the 384 cattle isolates that grouped into 38 profiles and 261 wild boar isolates that collapsed into 30 profiles, with 17 profiles common to the three host species (Fig. 6). The discriminatory power presents a differential evolution through time periods, and the underlying overall discriminatory power ranged from 0.92 in cattle isolates to 0.88 in red deer (Fig. 6).

Frequencies of spoligotyping profiles vary across host species, SB0121 (17.7%) is the most frequent in cattle, SB0122 (21.8%) in red deer and SB1174 (21.8%) in wild boar. The spoligotypes SB0122, SB1174 and SB1264 are the top three most common in the wildlife hosts, only with variable frequencies. When considering the percentages of the six most common profiles, per host species, the increase of the cumulative effect, through time, only occurred in wildlife datasets. The contribution for cattle dataset is stable, around 55%; while for wild boar it ranges from 61 to 76% and for red deer from 52 to 85%, from period 1 to 3, respectively.

Positive and significant relationships were established with all host species, being one profile (SB1264) associated to both wild boar and red deer (*p*-value < 0.01) (Supplementary Table S6). Red deer established positive associations with SB0122, SB1195 and SB1264; wild boar was associated with SB1174, SB1257, SB1264 and SB1265; and cattle with SB0119, SB0121, SB0140, SB0295, SB1090 and SB1172 (*p*-value < 0.01).

Complete MIRU-VNTR profiles included 194 isolates from cattle grouped into 100 MIRU types, 138 from red deer clustered into 55 MIRU types and 115 from wild boar into 58 MIRU types (Supplementary Fig. S4). Seventeen MIRU types were common to three host species, representing 45% of the global dataset (Supplementary Fig. S4). The M34, M225 and M261 types were most commonly reported in specific host species datasets, M34 (11.8%) in cattle, M225 (13.9%) in wild boar and M225 and M261 (12.3%) in red deer.

With an overall discriminatory power of 0.96, the wildlife datasets were approximately as diverse as cattle’ (D = 0.97). ETR-A was the *locus* registering the highest allelic diversity for all host species, while MIRU4 was the least diverse *locus* for cattle, MIRU26 for red deer and QUB11a for wild boar.

Fifteen genotypic profiles were shared by the three host species, whereas the number of shared genotypic profiles between pairs of host species is equal or inferior to 10.

### Network analysis

**Figure 7:**
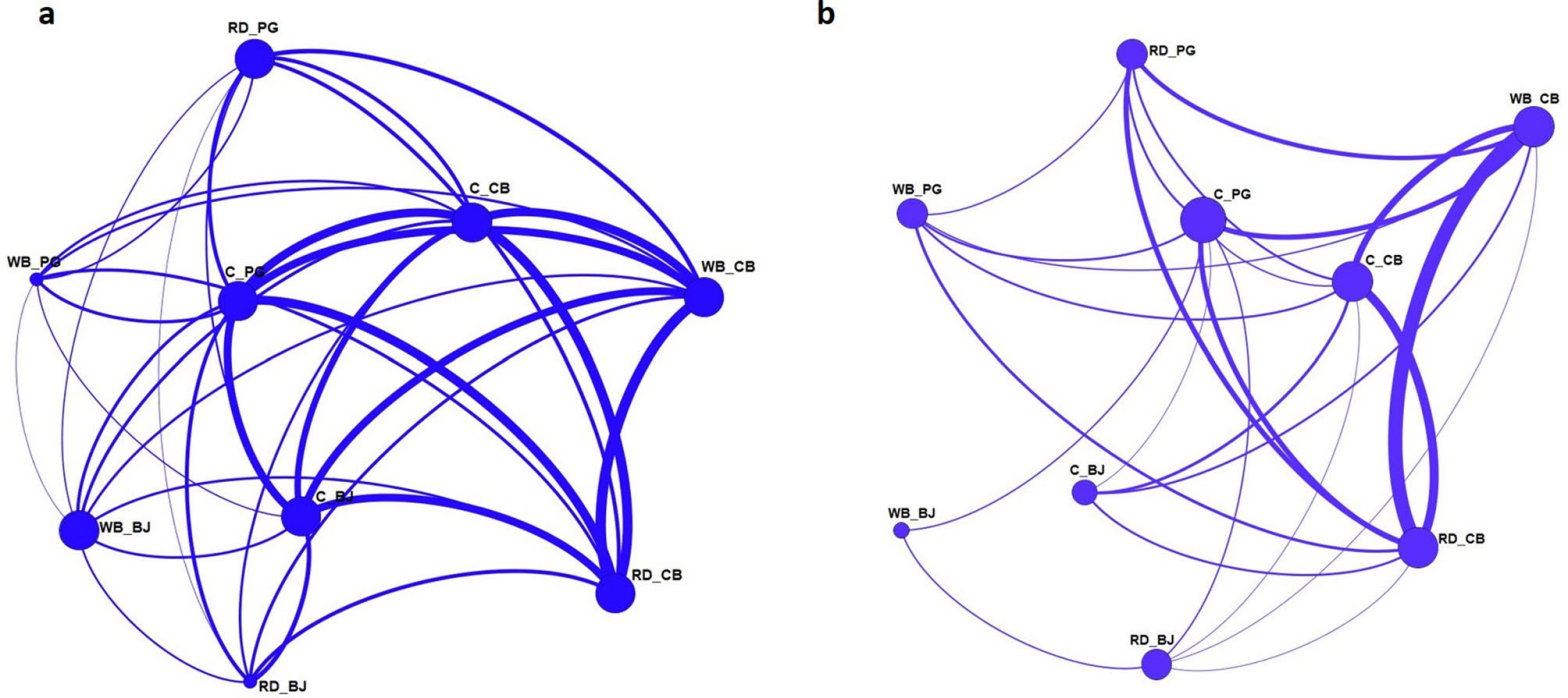
Global spoligotyping (**a**) and genotypic profiles (**b**) networks. The nodes represent the different host-geographic region pairs (RD, red deer; WB, wild boar; C, cattle—BJ, Beja; PG, Portalegre; CB, Castelo Branco) and the width of lines between them is proportional to the number of shared spoligotyping/genotypic profiles. For the spoligotyping-based network, the shared profiles varied between one and 16, while for the genotypic profile network the variation is from one to 19. The networks were generated with Gephi (version 0.9.2, https://gephi.org/).

A network analysis was performed to get a visual image of the strength of relationships established within each host-geographic region pair, using as connection lines the number of shared spoligotyping or genotypic profiles (Fig. 7a,b). Overall, results highlighted a strong regional spoligotyping network, linking the three host species within Castelo Branco and between Castelo Branco and Portalegre, with approximately half of the connections representing more than 12 shared spoligotyping profiles. Second, the high number and strength of epidemiological connections involving cattle, across and within all regions, is particularly impressive when considering spoligotyping profiles as linking criteria. In fact, in the spoligotyping network, all connections, with only one exception, represent over ten shared profiles. However, when considering genotypic profiles, the higher connection strengths (> 10 shared genotypic profiles) are exclusive of Castelo Branco and the cattle connections are established with an inferior number of profiles (< 5 genotypic profiles).

### Epidemiological tracing within livestock herds and officially-delimited hunting areas

Information concerning the herd of origin was available for 93% (*n* = 356) of cattle specimens, while the identity of officially-delimited hunting areas was obtainable for 86% (*n* = 479) of wildlife animals.

Criteria were established to discriminate two epidemiologically distinctive situations, small outbreaks and chronically infected scenarios. A chronically infected herd/hunting area was defined by the recovery of three or more *M. bovis* isolates in different years (consecutive or not). According to this, 48 cases, 19 in herds and 29 in hunting areas were identified. In the case of small outbreaks, the cases were confined to a single year, with indication of 14 events, being the majority (*n* = 12) in herds. The livestock herd with largest outbreaks account for 19 *M. bovis* and five spoligotyping profiles, as well as five genotypic profiles, while the hunting area with more isolates recovered registers 70 *M. bovis* isolates distributed across 15 spoligotypes and 23 genotypic profiles (Supplementary Fig. S5).

Globally, *M. bovis* isolates recovery was higher in hunting areas, when considering chronically infected scenarios, while the reverse situation was registered if small outbreaks are considered.

A detailed analysis of livestock herds (*n* = 4) and officially-delimited hunting areas (*n* = 12) with more than 10 *M. bovis* isolates recovered was performed (Supplementary Fig. S5). The outbreaks in livestock herds were all heterogeneous, with the existence of spoligotyping profiles common to two or more years, consecutive or not, and also with common genotypic profiles, but only in consecutive years. However, when considering officially-delimited hunting areas, the outbreaks are more complex, extending over larger time periods and with *M. bovis* recovered from the two wild host species. So, as expected, even when comparing outbreaks with the same number of isolates, the total of spoligotyping/genotypic profiles generated is higher in hunting areas, and two different situations can be described: specific genotypic profiles regularly detected throughout the outbreaks and particular genotypic profiles occasionally detected in a specific time period.

### Phylogenetic inferences of *M. bovis* isolates by MST

**Figure 8:**
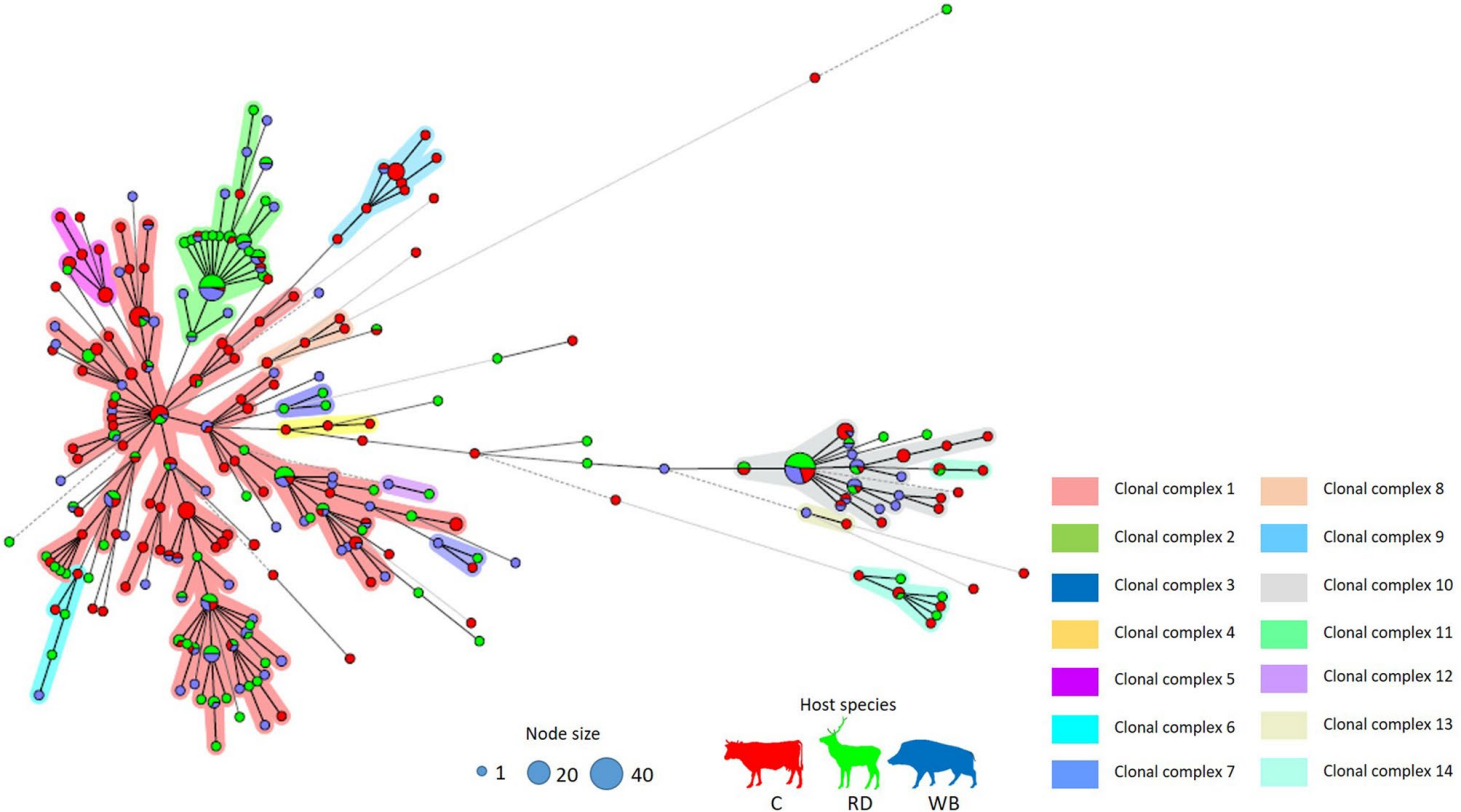
MST illustrating the evolutionary relationships among *M. bovis* isolates (*n* = 487) based on combined spoligotyping and 8-*loci* MIRU-VNTR data, using single *locus* variant analysis. Circle size is proportional to the number of isolates within each group; nodes are colored by host species (red for cattle (C), green for red deer (RD) and blue for wild boar (WB)); and the different clonal complexes are identified by different colors. The complexity of the lines denotes the number of differences in spoligo-MIRU type profile between two nodes: solid lines (1, 2 or 3 differences), gray dashed lines (4 differences) and gray dotted lines (5 or more differences). Profiles with double alleles were not included in this analysis. MST was generated with Bionumerics (version 6.6, http://www.applied-maths.com/bionumerics).

The majority of isolates (*n* = 428; 87.9%) were grouped into one of 14 clonal complexes (CC), with the largest complex encompassing 45% of isolates (*n* = 220) (Fig. 8). Some clonal complexes were specific to a single host species, while others were geographically clustered. The clonal complexes 4, 5 and 8 were exclusive of cattle, while CC 3 only encompasses red deer (Fig. 8). Geographic segregation is evident for clonal complexes CC 3, CC 6 and CC 12, only present in Castelo Branco; and for CC 5, exclusive for Beja isolates (Supplementary Fig. S6).

Clonal complexes 2 and 10, and two largest branches of CC 1, displayed a star-like shape typical of expanding populations^41^, with high-frequency central nodes surrounded by diffusing layers of variants (Fig. 8).

Downsizing the analysis to geographic region, identified 11 CC in Castelo Branco, eight in Portalegre and four in Beja (Supplementary Fig. S7). Central nodes of the largest three complexes in Castelo Branco are shared by two or three host species and display a structure coincident with an expanding population (Supplementary Fig. S7). In Portalegre, this is also evident for one clonal complex, while in Beja none of the defined complexes exhibit this architectural arrangement (Supplementary Fig. S7).

### Bayesian population structure

**Figure 9:**
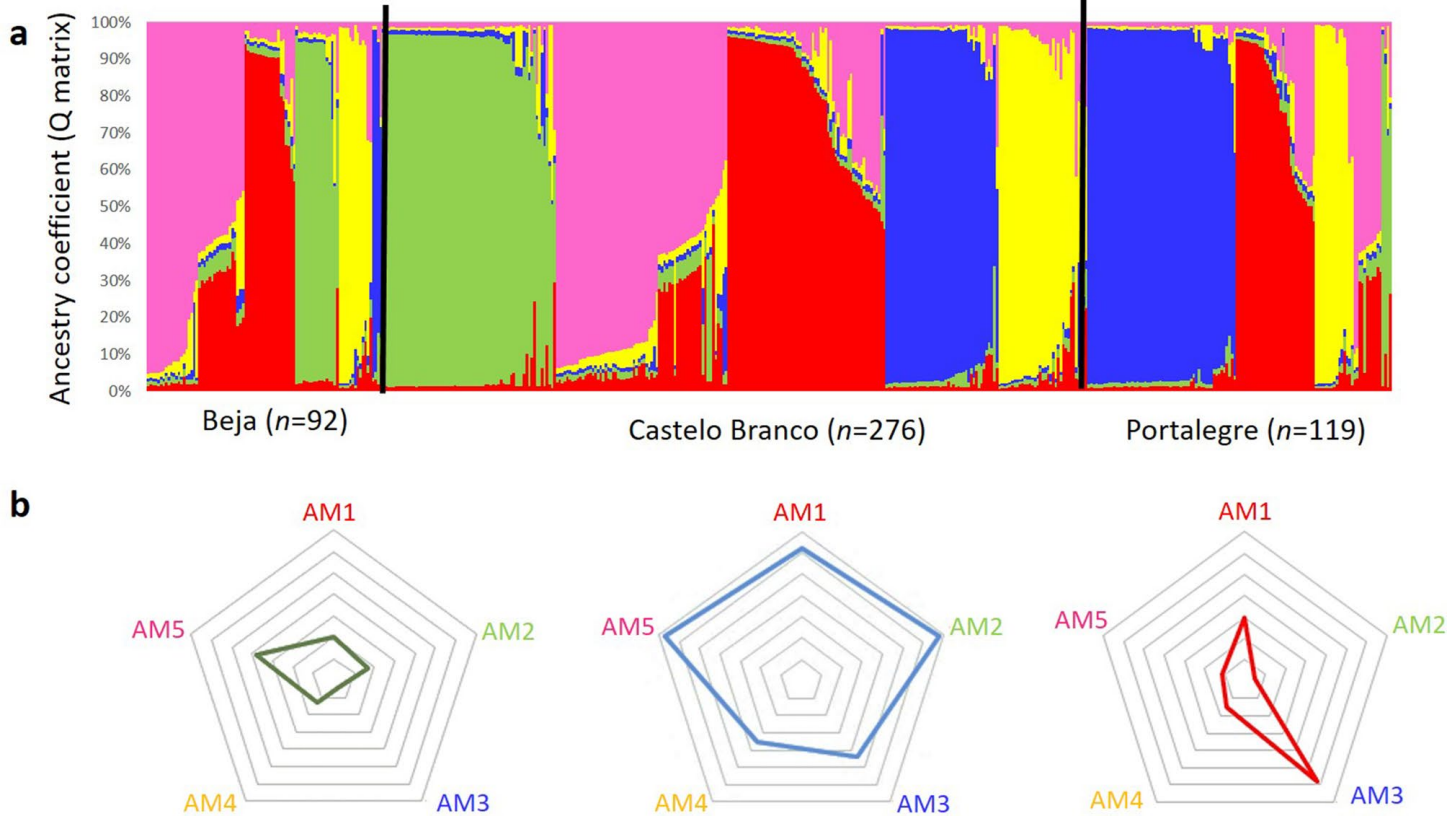
Bayesian clustering analysis, with detailed ancestry coefficient map: (**a**) distribution of *M. bovis* isolates pertaining to each hypothetical ancestral population (AM), (**b**) *per* geographic region.

**Figure 10:**
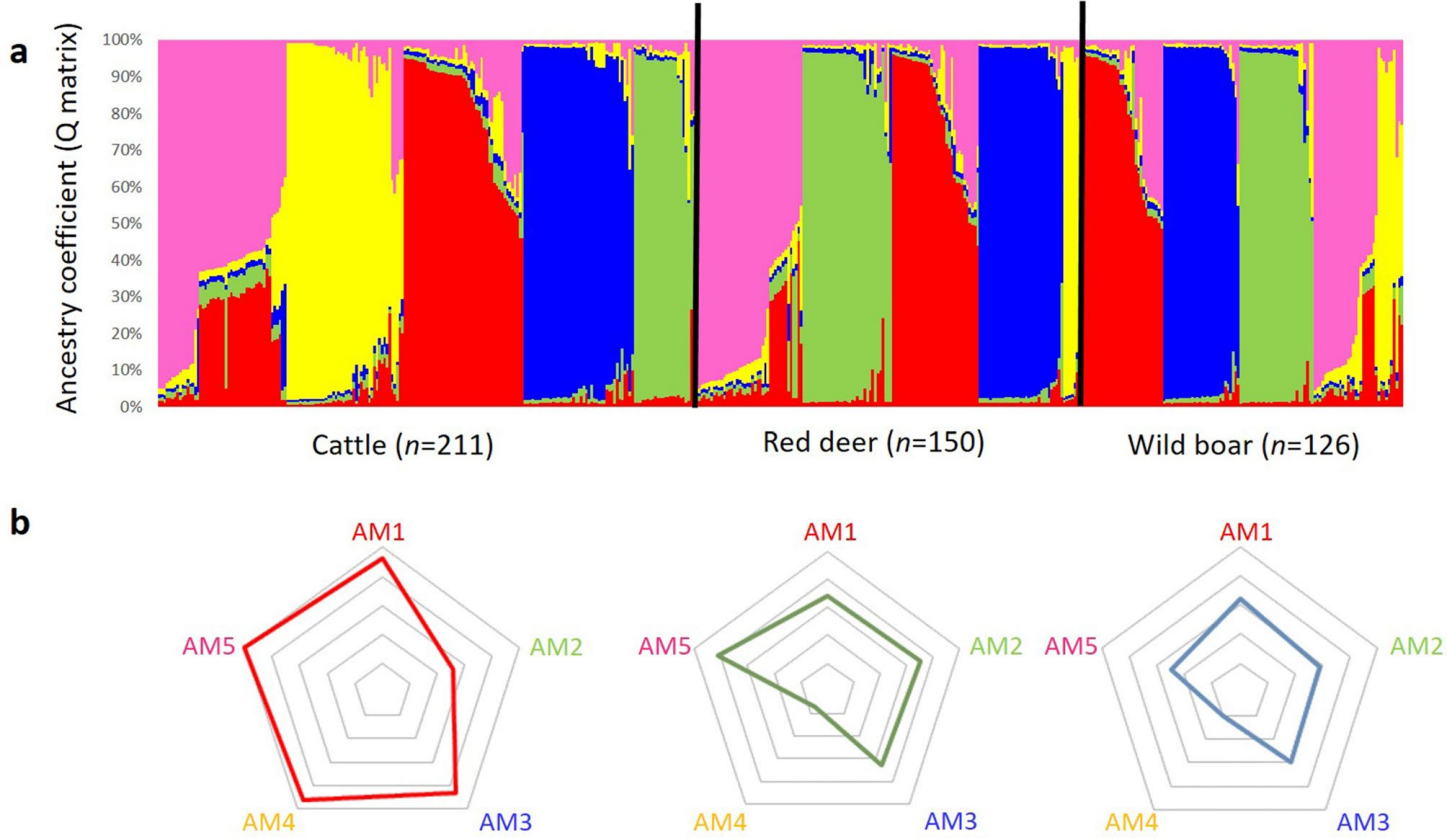
Bayesian cluster analysis, with detailed ancestry coefficient map: (**a**) distribution of *M. bovis* isolates pertaining to each hypothetical ancestral population (AM), (**b**) *per* host species.

The Bayesian clustering approach suggested the existence of K = 5 ancestral *M. bovis* (AM) populations (Supplementary Fig. S8), that were named ancestral *M. bovis* 1 to *M. bovis* 5 ancestral populations (AM1 to AM5) (Figs. 9a, 10a).

The spatial analysis of these five Bayesian-inferred ancestral populations revealed a contrasted geographical distribution (Fig. 9b, Supplementary Table S7): populations AM1, AM2, AM4 and AM5 are predominant in Castelo Branco, while AM3 is more evenly distributed between Castelo Branco and Portalegre. Moreover, population AM2 and AM3 are underrepresented in Portalegre and Beja, respectively (Fig. 9, Supplementary Table S7).

Host analysis (Fig. 10b, Supplementary Table S7) highlighted a tropism of AM4 population for cattle, while the other ancestral populations were more evenly distributed across different host species. This structure was further confirmed by performing a new MST analysis of *M. bovis* isolates, pre-labelled as populations AM1 to AM5 (Supplementary Fig. S9). Results were very congruent between both approaches, notably for populations AM2 and AM3, that revealed to be very well structured.

The geographic adaptation of ancestral populations was marked, with each geographic region evidencing positive and negative associations (p-value < 0.01) (Supplementary Table S8). Castelo Branco evidenced a positive association (p-value < 0.01) with population AM2, Portalegre with AM3 and Beja with AM5 (Fig. 9b). The negative associations were established at a *p*-value < 0.01 between population AM3 and Castelo Branco and Beja, and populations AM2 and AM5 with Portalegre (*p*-value < 0.01) (Fig. 9b).

Concerning host species, only cattle revealed a positive association with population AM4, while AM2 was negatively linked with cattle and AM4 with red deer (Fig. 10b).

### Genetic characteristics of AM1 to AM5 populations

When focusing on markers driving the structure of *M. bovis* populations, the *locus* with a higher individual allelic diversity (h) could be defined as QUB11a for populations AM2 and AM4, ETR-A for AM1, VNTR3232 for AM3 and ETR-C for AM5. MIRU4 was the least diverse *locus* in all populations, with the exception of AM1, for which QUB11b was the least diverse.

The mean allelic richness analysis revealed that AM4 holds the highest mean allelic richness, while AM5 reaches the lowest value.

## Discussion

Understanding the transmission dynamics of *M. bovis* circulating in an ecosystem and the role that each host species exerts in the spread and maintenance of the pathogen is necessary to improve existing control measures. The molecular methods implemented in this work thus granted an accurate monitoring of *M. bovis* genotypes over a 15-year period and a better understanding of the regional and local dynamics, such as small outbreaks and chronically infected scenarios both in herds and hunting estates. Spoligotyping generated a total of 64 profiles from three hotspot areas and a D value (0.92) similar to the global mean assessed a few years ago in a nationwide survey, in mainland Portugal (D = 0.93) by previous work^7^. SB0121 was previously reported as the most common in Portugal^7,42^, as well as in Spain^43–45^, and the third most frequent in France^24^. In this work, SB0121 remains the most common in cattle and the third for the entire dataset. About half of the identified spoligotypes, including the most common, were also previously reported in bordering Spain^43,45–48^. In fact, a recent work by García-Jiménez et al*.*^45^, in Extremadura region (that borders Castelo Branco and Portalegre districts), evidenced shared spoligotypes among livestock and wildlife species. The high agreement of spoligotyping profiles reported by Portugal and Spain could be related with historical backgrounds, that include long trade relationships, in addition to wild animal migrations throughout the border and disease expansion from the core Iberian cluster of disease.

Down-sizing this analysis to the administrative regions and to the host species level, Beja (the region where the number of cattle and wildlife isolates is more unbalanced) and cattle, subsist as the scenarios in which the reduction in genetic variability is less evident. In Beja and cattle sub-datasets, the evolution of the cumulative influence of the most prevalent spoligotyping profiles (that is the cumulative proportion of most common profiles), across temporal periods, reaches around 50% in 2016. The decrease of genetic variability over time is in agreement with other long-term studies performed in livestock-wildlife epidemiological settings^49^, and with the scenario concerning *M. tuberculosis* complex members’ population structure and evolution. Clonality reflects evolution processes dominated by reductions in diversity caused by population bottlenecks and selective sweeps^50^. The implementation of test and cull eradication programs in cattle enables continuous surveillance over these populations, turning disease outbreaks smaller and geographically stratified, so that decrease in genotypic diversity is smaller, when compared with wildlife populations with sustained genotype flow in their vital areas.

Globally, 26 spoligotyping profiles, representing 94.7% of total *M. bovis* isolates, were found in two or three host species. Diversity calculations within each species offered insight into their relative contribution to the epidemiology of animal TB. The occurrence of host exclusive spoligotyping profiles was suggested as an indicator to assess the importance of maintenance hosts^51^ and specific host profiles were registered in all three species. The greater commonality of genotypes shared between red deer and cattle give an emphasis to the role of red deer at the domestic-wildlife interface. Previous studies on social behaviour and spatio-temporal interactions at the cattle-wildlife interface, in a similar ecosystem in south-central Spain, reported that red deer daily activity patterns slightly overlap with cattle^11,52^, making direct transmission events more likely to occur, when comparing with wild boar that present a different daily activity pattern as a crepuscular or nocturnal species, turning direct transmission unlikely. However, a recent spatial interaction study performed in Spain revealed that environmental conditions during summer and autumn, when water and food supplies are reduced, can promote encounters of wild boar and cattle^53^.

The spatial–temporal analysis applied to our dataset reinforces the notion that the cattle-wildlife interaction is important, since the two significant clusters obtained were formed by the three host species, however in different associated proportions and relative risk, pointing out for two remarkably different epidemiological scenarios. A first epidemiological setting located in Beja, occurring between the 2004 and 2010 period that highlighted a higher associated risk for cattle; and a second, posterior scenario, positioned in Castelo Branco, underlined in the 2012–2016 period, in which wildlife species, particularly wild boar, present higher relative risk. This temporal and spatial stratified situation cannot be dissociated from the characteristics of our dataset, with wildlife sampling only occurring in a regular way from 2011 onward, being underrepresented up until that year and being strongly dependent upon hunting actions. Not all municipalities did regular, systematic work examining hunter-harvested big game. Also, the hunting bags vary across regions and year. Therefore, these results need to be analysed with caution and a regular update should be performed in the coming years to understand the clustering behaviour.

Molecular data reinforce and support livestock-wildlife and wildlife-wildlife transmission, either by direct or indirect ways, highlighting wildlife as keystone hosts in animal TB dynamics and supporting the general notion that wildlife may negatively contribute to the efficiency of TB eradication programs in cattle. Knowing that homoplasy of spoligotyping and MIRU typing^54,55^ can impact on transmission inference, a combined approach (i.e. with spoligotyping and MIRU-VNTR panel) was applied to minimize those risks and to confer more discriminatory power and accuracy to our conclusions. A panel of 8 *loci* was selected, not only because it was previously applied to Portuguese isolates with high discriminatory capacity, but also because a higher number of *loci* decrease homoplasy risks^55^. Indeed, the application of this typing technique enabled a more refined discrimination of isolates.

On a regional level, the specific discriminatory capacity obtained for each *locus* is not in agreement with previous data from Portugal^7,20^, and is uneven across geographic regions. The variation in allelic diversity between geographic regions and the similarities observed across adjacent geographic regions suggest a contrasting evolutionary history of these *loci* across regions, as well as the biogeographic segregation of *M. bovis* subpopulations and local processes of evolution. In fact, the network built upon the genotypic profiles revealed strong links established within geographic regions and not in between. Work performed in Northern Ireland also demonstrated the geographical structure of MIRU profiles obtained from cattle isolates, pinpointing the importance of local dynamics^56^.

The uniformity of genotypic profiles found in cattle and wildlife, within the same spatiotemporal context, reinforces epidemiological connections and suggests intra- and interspecies transmission, as already described in Iberian multi-host systems (including, not only red deer and wild boar^48^, but also badgers^57^); in Italy^58^ and Canada^59^. Some genotypic profiles are restricted in time and space, such as SB0121-M219 that is shared by cattle and red deer in Castelo Branco, during 2008; or SB1174-M260 that is common to three host species in Castelo Branco, during 2016. In contrast, others are actively in circulation during long time periods, for example SB1190-M34 that was detected within the three hosts species between 2009 to 2014, both in Beja and Portalegre (see supplementary information). Interestingly, the genotypic profiles common to more than one host species are the ones that were maintained in circulation for longer periods of time, reinforcing the importance of this multi-host system for *M. bovis* persistence and transmission, and possibly reflecting a better strain adaptation to the environment and/or to the host. Similar results were observed in France, where the local spread of specific genotypes was registered in regions with wildlife TB cases^24^. When downsizing to local level of livestock herds and hunting areas, the epidemiological scenario could be evaluated in closer detail, with the identification of different genotypic profiles over the years and within the same year, supporting different sources of infection. The presence of wildlife, interaction between cattle-wildlife^11,60,61^ and environmental contamination^4,62^ could exert a strong influence in herd TB dynamics, as suggested by the genotypic diversity encountered, even in small outbreaks, with the identification of up to six genotypic profiles within the same year. The presence of wildlife, namely spatial distribution, densities and artificial management conditions, have been associated as risk factors for TB in cattle. The relationship between densities of badgers, brushtail possums and wild boar were already associated with cattle TB in the UK, New Zealand and Iberian Peninsula, respectively^13,63,64^. These parameters are starting to be included in modelling approaches to simulate the efficiency of different TB surveillance and control systems^65,66^.

Plus, cattle movement between herds due to trading, breeding activities, extensive production systems and proximity to hunting zones can also have an important impact in *M. bovis* transmission, as indicated in other works^67–70^. Indeed, cattle trading is often studied as a driving force of TB introduction into cattle herds^71–73^. Chronically infected officially-delimited hunting areas also evidenced a huge genotypic diversity, being the most problematic epidemiological situations located in Castelo Branco. The identification of officially-delimited hunting areas with up to 23 genotypic profiles suggests that translocations of wild ungulates for hunting purposes may change the distribution map of TB strains and highlights the disease risks that occur if appropriate veterinary control measures are not implemented. Recrudescence of specific genotypes in a hunting area were also evident by longitudinal analysis, suggesting that some genotypes may be better adapted for multi-host infection or better detected by conventional testing methods. Moreover, the presence of super-shedders in badger populations from the UK^74^, and red deer and wild boar from Portugal^14^ have been reported possibly contributing to the persistence of specific genotypes. Concomitantly with this intricate transmission scenario, concomitant infection of individual hosts with two *M. bovis* genotypic variants identified by the presence of double alleles. This situation suggests two possible scenarios, co-infection with different strains acquired by the host in a single occasion or on separated time points, which is a very plausible scenario if one considers the epidemiological situation just described up above, with different genotypic profiles circulating even in small outbreaks; or the occurrence of an in vivo microevolutionary event on the founder strain. In the majority of cases, double alleles were detected only in one *loci*, with special prevalence in VNTR3232, supporting the hypothesis of microevolution phenomena, once the infection of one host by two very similar genotypic strains is not that likely in a scenario with high genotypic diversity. However, in a smaller group of strains, double alleles were detected in two to four *loci* of the same genome, which would tend to be the result of a co-infection by different strains. The smaller group of cases in which this occurred was restricted to wildlife hosts, so it might be related with their increased mobility for food and water supplies that promote contact with different *M. bovis* contamination sources^44,75,76^. Recent work conducted by our research group with *M. caprae* isolates also revealed the presence of double alleles, exclusively in MIRU4^32^, which contrasts with data from this work, that evidenced a higher prevalence in VNTR3232 but not exclusive to this *locus*. Either way, this is a subject that deserves further investigation in the future. Furthermore, MIRU-VNTR just focuses on a small fraction of the genome and has limited resolution to capture microevolution events in other genomic regions. The identification of particularities in this work regarding chronically infected scenarios at the livestock-wildlife interface in Portugal will inform and guide a more cost-effective, risk-based, TB prevention and control strategy in these geographic regions. Given that the consistency of genotypic profiles identified in the same herd/officially-delimited hunting area in different time periods suggests reactivation of the disease, the failure to abolish the infection source is underlined, requiring more stringent surveillance. In contrast, epidemiological scenarios with multiple profiles could be related with disease re-introduction by artificial management and translocations/movements, once again calling for action.

The *M. bovis* population structure defined through minimum spanning trees and the Bayesian clustering algorithm implemented in STRUCTURE reflect the strain diversity across the three districts. Forty-five percent of the *M. bovis* dataset is contained in a single clonal complex. The fact that a substantial part of genotypes was developed from single or double *locus* variations upon basal types highlights a plausible clonal structure that is consistent with the expansion of a founder strain. The geographic stratification analysis of genotypic composition performed per district demark a central node in Beja exclusively composed by cattle genotypes (SB0121-derived profiles) that then irradiate to deer’ and wild boar’ strains; cattle and red deer strains at the expanding central node of Portalegre; and a mixed central node in Castelo Branco that is constituted by strains from cattle and wildlife hosts that suggests an ancestral common origin. Herewith, Beja was pointed out as the most ancient population, while Castelo Branco and Portalegre appear to be expanding, a very interesting finding that correlates with the central node composition of stratified MST, considering different host species in their composition.

Currently, five *M. bovis* clonal complexes have been described in the literature, European 1 (Eu1), 2 (Eu2) and 3 (Eu3), and African 1 (Af1) and 2 (Af2)^77–81^. Forty-two spoligotyping profiles accounting with 64.5% of isolates within this dataset present a deletion in spacer 21 (specific of European 2), while a smaller proportion of profiles exhibit deletion in spacer 11 (specific of European 1); in spacers 3 to 7 (specific of African 2) and present SB0120 (representative profile of European 3). The analysis of specific gene profiling (e.g. SNP analysis of *guaA* for Eu2) was not performed, so that grouping of *M. bovis* isolates in the defined clonal complexes is not possible, but the majority are candidates to belong to clonal complex European 2. This indication is in agreement with the known geographic specificities of clonal complexes distribution, that suggest that the majority of *M. bovis* from the Iberian Peninsula are included in clonal complex European 2^77^. Bayesian clustering inferences provided evidence for geographic specificity of defined *M. bovis* ancestral populations and statistically significant correlations with cattle. Although there are commonalities across the three regions, *M. bovis* adapted to each hotspot area present specific genotypic characteristics, which is in agreement with differential prevalence rates of the circulating strains in a region and also with the distinctive discriminatory capacity of each MIRU-VNTR *locus* across regions. These results suggest that genotypic differences are more dependent on location than host species, an information that can be useful when planning control measures for each epidemiological scenario.

Intersecting genotypic data with information from the studies published so far, concerning characteristics of infection, the way animals use the environment and the interactions they establish can be key in determining the local risk for disease transmission and maintenance. Previously published data concerning TB lesions revealed a high frequency of red deer presenting multiple non-calcified lesions at the abdomen and thorax, whereas most lesions in wild boar are single and calcified^10,13^. Multiple lesions are suggestive of higher susceptibility carrying a higher potential for excretion of the pathogen, in parallel with lesion location and structure. In one of the studied areas, Castelo Branco district, previous work revealed that density of red deer was consistently the most relevant and significant factor influencing *M. bovis* infection in red deer and wild boar^13^.

The population structure we came across with was further confirmed by performing new MST analyses with isolates classified as population AM1 to AM5. A good agreement between Bayesian clustering inference and MST analysis was established, pronouncing the value of microsatellite genetic structure that is almost as robust as combined Spoligo/MIRU-VNTR phylogeny. The agreement among our different phylogenetic approaches supports the clonal evolution model of *M. tuberculosis* complex members^82^. Using allelic richness as a criterion of diversification, population AM4 seems to be more ancient. Interestingly, this ancestral population is statistically associated with cattle, a host species described to be *M. bovis* main host species, and Beja, providing clues on the who infected whom, cattle or wildlife, question.

While ongoing surveillance has provided valuable information on the prevalence and spatial occurrence of TB in areas where wild boar and red deer are sympatric, the possibility of massive whole genome sequencing (WGS) at low cost could revolutionize our current view of TB epidemiology in Iberia. The ability to observe point mutations in *M. bovis* genome could allow tracking transmission down to the individual or farm level, and to infer population level contact structure characteristics. Specifically, WGS could identify genetic clustering of isolates indicative of intra-species transmission, and so potentially estimate how much inter-species transmission there is amongst sampled wild and cattle populations, as well contribution of environmental reservoirs. Whole genome sequencing, contrary to spoligotyping and MIRU-VNTR, would enable the identification of sequence variations across the entire genome and single nucleotide polymorphisms could be used to study the relationships among isolates, with the advantage of presenting low reverse mutation rates and homoplasy indexes, supplanting several of the typing techniques limitations.

## Concluding remarks

In this work, spoligotyping and MIRU-VNTR were applied for the molecular characterization of *M. bovis* longitudinally obtained during a 15-year period across TB hotspot areas in Portugal. Together, these techniques provided solid information concerning genotyping diversity and updated the characteristics of *M. bovis* isolates circulating across host species, within populations and across geographic regions. Geographic specificities revealed by MST and Bayesian clustering inference analyses assign Beja as the more ancient population and epidemiologically link contiguous districts, Castelo Branco and Portalegre, with locally segregated but expanding *M. bovis* populations that seem to be better adapted for multi-host infection.

Results support the hypothesis of a good TB maintenance community across the three hosts species and locations under analysis, and the confirmation of intraspecific and interspecific transmission, namely at the livestock-wildlife interface, pointing out an important reservoir role for cattle, probable direct transmission to red deer, but also a prominent role for wild boar as a reservoir, recommending the need to harsh control on cattle and to further consider wildlife behaviour and abundance in the adjustment of the TB eradication program in Portugal.

## Supplementary information

Supplementary Information 1.

Supplementary Information 2.
