# Author Correction: Long-term molecular surveillance provides clues on a cattle origin for *Mycobacterium bovis* in Portugal

**DOI:** 10.1038/s41598-021-86047-y

**Published:** 2021-03-24

**Authors:** Ana C. Reis, Rogério Tenreiro, Teresa Albuquerque, Ana Botelho, Mónica V. Cunha

**Affiliations:** 1 Centre for Ecology, Evolution and Environmental Changes (cE3c), Faculdade de Ciências da Universidade de Lisboa, Campo Grande, 1749-016 Lisboa, Portugal; 2 Biosystems & Integrative Sciences Institute (BioISI), Faculdade de Ciências da Universidade de Lisboa, Lisboa, Portugal; 3 National Institute for Agrarian and Veterinary Research (INIAV IP), Oeiras, Portugal

Correction to: *Scientific Reports* 10.1038/s41598-020-77713-8, Published online 30 November 2020

This Article contains a typographical error in the Acknowledgements section.

“This work was also supported by strategic funding to cE3c and BioISI Research Units (UID/BIA/00329/2020 and UID/Multi/04046/2020).”

should read:

“This work was also supported by strategic funding to cE3c and BioISI Research Units (UIDB/00329/2020 and UIDB/04046/2020).”

