# Supplementary material for: Long-term molecular surveillance provides clues on a cattle origin for Mycobacterium bovis in Portugal
Source: Sci Rep. 2020 Nov 30;10:20856. doi: 10.1038/s41598-020-77713-8 (PMC7705689; doi:10.1038/s41598-020-77713-8)
Supplement: Supplementary file 1 — Supplementary Information 1. [file 41598_2020_77713_MOESM1_ESM.docx]

**Long-term molecular surveillance provides clues on a cattle origin for *Mycobacterium bovis* in Portugal**

Ana C. Reis^1,2^, Rogério Tenreiro^2^, Teresa Albuquerque^3^, Ana Botelho^3^, Mónica V. Cunha^1,2*^

^1^Centre for Ecology, Evolution and Environmental Changes (cE3c), Faculdade de Ciências da Universidade de Lisboa, Lisboa, Portugal

^2^Biosystems & Integrative Sciences Institute (BioISI), Faculdade de Ciências da Universidade de Lisboa, Lisboa, Portugal

^3^INIAV, IP- National Institute for Agrarian and Veterinary Research, Oeiras, Portugal

*Correspondence: mscunha@fc.ul.pt; Tel.: +351 217 500 000;

Faculdade de Ciências da Universidade de Lisboa, Campo Grande, 1749-016 Lisboa, Portugal

**Supplementary table S1.** Molecular differentiation of *M. bovis* isolates by spoligotyping.

| **Spoligotyping profile** | **Prevalence (%)** | **Host species** | **Geographic region** | **Year(s) of isolation** | **Spoligotyping 43 spacers profiles** |
| --- | --- | --- | --- | --- | --- |
| SB0119 (*n*=57) | 6.0 | C, RD, WB | CB, PG, BJ | 2003, 2006-2010, 2013, 2014, 2016 | ⬛⬛⬜⬛⬛⬛⬛⬛⬜⬛⬛⬛⬛⬛⬜⬜⬛⬛⬛⬛⬜⬛⬛⬛⬛⬛⬛⬛⬛⬛⬛⬛⬛⬛⬛⬛⬛⬛⬜⬜⬜⬜⬜ |
| SB0120 (*n*=24) | 2.5 | C, RD, WB | CB, PG, BJ | 2004, 2006-2010, 2012-2016 | ⬛⬛⬜⬛⬛⬛⬛⬛⬜⬛⬛⬛⬛⬛⬛⬜⬛⬛⬛⬛⬛⬛⬛⬛⬛⬛⬛⬛⬛⬛⬛⬛⬛⬛⬛⬛⬛⬛⬜⬜⬜⬜⬜ |
| SB0121 (*n*=111) | 11.7 | C, RD, WB | CB, PG, BJ | 2002, 2004, 2006-2016 | ⬛⬛⬜⬛⬛⬛⬛⬛⬜⬛⬛⬛⬛⬛⬛⬜⬛⬛⬛⬛⬜⬛⬛⬛⬛⬛⬛⬛⬛⬛⬛⬛⬛⬛⬛⬛⬛⬛⬜⬜⬜⬜⬜ |
| SB0122 (*n*=123) | 13.0 | C, RD, WB | CB, PG, BJ | 2004, 2005, 2007-2016 | ⬜⬛⬜⬛⬛⬛⬛⬛⬜⬛⬛⬛⬛⬛⬛⬜⬛⬛⬛⬛⬜⬛⬛⬛⬛⬛⬛⬛⬛⬛⬛⬛⬛⬛⬛⬛⬛⬛⬜⬜⬜⬜⬜ |
| SB0124 (*n*=2) | 0.2 | C | PG, BJ | 2014 | ⬛⬜⬜⬜⬛⬛⬛⬛⬜⬛⬛⬛⬛⬛⬛⬜⬛⬛⬛⬛⬜⬛⬛⬛⬛⬛⬛⬛⬛⬛⬛⬛⬛⬛⬛⬛⬛⬛⬜⬜⬜⬜⬜ |
| SB0133 (*n*=1) | 0.1 | C | BJ | 2009 | ⬛⬛⬜⬜⬜⬜⬜⬛⬜⬛⬛⬛⬛⬛⬛⬜⬛⬛⬛⬛⬛⬛⬛⬛⬛⬛⬛⬛⬛⬛⬛⬛⬛⬛⬛⬛⬛⬛⬜⬜⬜⬜⬜ |
| SB0134 (*n*=6) | 0.6 | C, RD, WB | CB, BJ | 2010,2011, 2014-2016 | ⬛⬛⬜⬜⬜⬛⬛⬛⬜⬛⬛⬛⬛⬛⬛⬜⬛⬛⬛⬛⬛⬛⬛⬛⬛⬛⬛⬛⬛⬛⬛⬛⬛⬛⬛⬛⬛⬛⬜⬜⬜⬜⬜ |
| SB0140 (*n*=26) | 2.7 | C, RD, WB | CB, PG, BJ | 2004, 2007-2011, 2013 | ⬛⬛⬜⬛⬛⬜⬛⬜⬜⬜⬜⬜⬛⬛⬛⬜⬛⬛⬛⬛⬛⬛⬛⬛⬛⬛⬛⬛⬛⬛⬛⬛⬛⬛⬛⬛⬛⬛⬜⬜⬜⬜⬜ |
| SB0152 (*n*=3) | 0.3 | C, RD | CB, PG | 2011, 2012, 2016 | ⬛⬜⬜⬜⬜⬜⬜⬜⬜⬜⬜⬜⬜⬜⬜⬜⬜⬜⬜⬜⬜⬛⬛⬛⬛⬛⬛⬛⬛⬛⬛⬛⬛⬛⬛⬛⬛⬛⬜⬜⬜⬜⬜ |
| SB0265 (*n*=75) | 7.9 | C, RD, WB | CB, PG, BJ | 2003, 2004, 2006-2016 | ⬛⬛⬜⬛⬛⬜⬛⬛⬜⬛⬛⬛⬛⬛⬛⬜⬛⬛⬛⬛⬜⬛⬛⬛⬛⬛⬛⬛⬛⬛⬛⬛⬛⬛⬛⬛⬛⬛⬜⬜⬜⬜⬜ |
| SB0269 (*n*=1) | 0.1 | RD | CB | 2009 | ⬛⬛⬜⬛⬛⬜⬛⬜⬜⬜⬜⬜⬛⬛⬜⬜⬛⬛⬛⬛⬛⬛⬛⬛⬛⬛⬛⬛⬛⬛⬛⬛⬛⬛⬛⬛⬛⬛⬜⬜⬜⬜⬜ |
| SB0294 (*n*=1) | 0.1 | WB | CB | 2008 | ⬛⬛⬜⬛⬛⬛⬛⬛⬜⬛⬛⬜⬛⬛⬛⬜⬛⬛⬛⬛⬜⬛⬛⬛⬛⬛⬛⬛⬛⬛⬛⬛⬛⬛⬛⬛⬛⬛⬜⬜⬜⬜⬜ |
| SB0295 (*n*=31) | 3.3 | C, RD, WB | CB, BJ | 2004, 2006, 2007, 2009, 2010, 2012, 2013, 2015, 2016 | ⬛⬛⬜⬛⬛⬛⬛⬛⬜⬛⬛⬛⬛⬛⬛⬜⬛⬛⬛⬛⬜⬛⬛⬛⬛⬛⬛⬛⬛⬛⬛⬛⬛⬛⬛⬛⬜⬛⬜⬜⬜⬜⬜ |
| SB0298 (*n*=1) | 0.1 | RD | CB | 2010 | ⬛⬛⬜⬛⬛⬜⬛⬜⬜⬜⬜⬜⬛⬛⬛⬜⬛⬛⬛⬛⬜⬛⬛⬛⬛⬛⬛⬛⬛⬛⬛⬛⬛⬛⬛⬛⬛⬛⬜⬜⬜⬜⬜ |
| SB0329 (*n*=1) | 0.1 | WB | CB | 2016 | ⬛⬛⬜⬛⬛⬛⬛⬛⬜⬛⬛⬛⬛⬛⬛⬜⬛⬛⬛⬛⬜⬛⬛⬛⬛⬛⬛⬛⬛⬛⬛⬛⬛⬛⬛⬛⬜⬜⬜⬜⬜⬜⬜ |
| SB0334 (*n*=1) | 0.1 | C | BJ | 2006 | ⬛⬜⬜⬛⬛⬛⬛⬛⬜⬛⬛⬛⬛⬜⬜⬜⬛⬛⬛⬛⬜⬛⬛⬛⬛⬛⬛⬛⬛⬛⬛⬛⬛⬛⬛⬛⬛⬛⬜⬜⬜⬜⬜ |
| SB0426 (*n*=1) | 0.1 | WB | CB | 2014 | ⬛⬛⬜⬜⬜⬛⬛⬛⬜⬛⬛⬛⬛⬛⬛⬜⬛⬛⬛⬛⬜⬛⬛⬛⬛⬛⬛⬛⬛⬛⬛⬛⬛⬛⬛⬛⬛⬛⬜⬜⬜⬜⬜ |
| SB0833 (*n*=1) | 0.1 | C | PG | 2014 | ⬛⬛⬜⬛⬛⬛⬛⬛⬜⬛⬛⬛⬛⬛⬛⬜⬛⬛⬛⬛⬛⬛⬛⬛⬛⬛⬛⬛⬛⬛⬜⬛⬛⬛⬛⬛⬛⬛⬜⬜⬜⬜⬜ |
| SB0848 (*n*=4) | 0.4 | C | CB | 2004 | ⬛⬛⬜⬛⬛⬛⬛⬛⬜⬛⬛⬛⬜⬛⬛⬜⬛⬛⬛⬛⬜⬛⬛⬛⬛⬛⬛⬛⬛⬛⬛⬛⬛⬛⬛⬛⬛⬛⬜⬜⬜⬜⬜ |
| SB0849 (*n*=1) | 0.1 | C | BJ | 2007 | ⬛⬛⬜⬛⬜⬛⬛⬛⬜⬛⬛⬛⬛⬛⬛⬜⬛⬛⬛⬛⬛⬛⬛⬛⬛⬛⬛⬛⬛⬛⬛⬛⬛⬛⬛⬛⬛⬛⬜⬜⬜⬜⬜ |
| SB0856 (*n*=9) | 0.9 | C, RD, WB | CB, BJ | 2007-2009, 2011, 2012, 2014, 2015 | ⬛⬛⬜⬛⬛⬛⬛⬛⬜⬛⬛⬛⬛⬛⬜⬜⬛⬛⬛⬛⬛⬛⬛⬛⬛⬛⬛⬛⬛⬛⬛⬛⬛⬛⬛⬛⬛⬛⬜⬜⬜⬜⬜ |
| SB0886 (*n*=1) | 0.1 | C | BJ | 2004 | ⬛⬛⬜⬛⬛⬛⬛⬛⬜⬛⬛⬛⬛⬛⬛⬜⬛⬛⬜⬛⬛⬛⬛⬛⬛⬛⬛⬛⬛⬛⬛⬜⬛⬛⬛⬛⬛⬛⬜⬜⬜⬜⬜ |
| SB0961 (*n*=1) | 0.1 | RD | PG | 2013 | ⬛⬜⬜⬛⬛⬛⬛⬛⬜⬛⬛⬛⬛⬛⬛⬜⬛⬛⬛⬛⬛⬛⬛⬛⬛⬛⬛⬛⬛⬛⬛⬛⬛⬛⬛⬛⬛⬛⬜⬜⬜⬜⬜ |
| SB1017 (*n*=1) | 0.1 | RD | CB | 2012 | ⬜⬜⬜⬛⬛⬛⬛⬛⬜⬛⬛⬛⬛⬛⬛⬜⬛⬛⬛⬛⬜⬛⬛⬛⬛⬛⬛⬛⬛⬛⬛⬛⬛⬛⬛⬛⬛⬛⬜⬜⬜⬜⬜ |
| SB1018 (*n*=6) | 0.6 | C, RD, WB | CB, BJ | 2007, 2008, 2012 | ⬛⬛⬜⬛⬛⬜⬛⬛⬜⬛⬛⬛⬛⬛⬜⬜⬛⬛⬛⬛⬜⬛⬛⬛⬛⬛⬛⬛⬛⬛⬛⬛⬛⬛⬛⬛⬛⬛⬜⬜⬜⬜⬜ |
| SB1053 (*n*=1) | 0.1 | RD | BJ | 2012 | ⬛⬜⬜⬛⬛⬛⬛⬛⬜⬛⬛⬛⬛⬛⬜⬜⬛⬛⬛⬛⬜⬛⬛⬛⬛⬛⬛⬛⬛⬛⬛⬛⬛⬛⬛⬛⬜⬛⬜⬜⬜⬜⬜ |
| SB1060 (*n*=2) | 0.2 | WB | CB | 2015 | ⬛⬜⬜⬛⬛⬛⬛⬛⬜⬛⬛⬛⬛⬛⬜⬜⬛⬛⬛⬛⬛⬛⬛⬛⬛⬛⬛⬛⬛⬛⬛⬛⬛⬛⬛⬛⬛⬛⬜⬜⬜⬜⬜ |
| SB1074 (*n*=1) | 0.1 | C | BJ | 2010 | ⬛⬛⬜⬜⬜⬛⬛⬛⬜⬜⬛⬛⬛⬜⬜⬜⬛⬛⬛⬛⬛⬛⬛⬛⬛⬛⬛⬛⬛⬛⬛⬛⬛⬛⬛⬛⬛⬛⬜⬜⬜⬜⬜ |
| SB1090 (*n*=27) | 2.8 | C, WB | CB, PG, BJ | 2007-2013 | ⬛⬛⬜⬛⬛⬛⬛⬛⬜⬛⬛⬛⬛⬛⬛⬜⬛⬛⬛⬛⬜⬜⬛⬛⬛⬛⬛⬛⬛⬛⬛⬛⬛⬛⬛⬛⬛⬛⬜⬜⬜⬜⬜ |
| SB1091 (*n*=1) | 0.1 | RD | CB | 2004 | ⬛⬛⬜⬛⬛⬛⬛⬛⬜⬛⬛⬛⬜⬛⬛⬜⬛⬛⬛⬛⬜⬛⬛⬛⬛⬛⬛⬛⬛⬛⬛⬛⬛⬛⬛⬛⬜⬛⬜⬜⬜⬜⬜ |
| SB1095 (*n*=6) | 0.6 | C, RD, WB | Castelo Branco | 2006, 2012, 2013 | ⬛⬜⬜⬛⬛⬛⬛⬛⬜⬛⬛⬛⬛⬛⬛⬜⬛⬛⬛⬛⬜⬛⬛⬛⬛⬛⬛⬛⬛⬛⬛⬛⬛⬛⬛⬛⬛⬛⬜⬜⬜⬜⬜ |
| SB1167 (*n*=5) | 0.5 | C, RD, WB | CB, PG | 2006, 2013, 2014 | ⬛⬛⬜⬛⬛⬛⬛⬛⬜⬛⬛⬛⬛⬛⬛⬜⬛⬛⬛⬛⬛⬛⬜⬛⬛⬛⬛⬛⬛⬛⬛⬛⬛⬛⬛⬛⬛⬛⬜⬜⬜⬜⬜ |
| SB1172 (*n*=14) | 1.5 | C, RD | CB, PG, BJ | 2004, 2005, 2007, 2008, 2011, 2013 | ⬛⬛⬜⬛⬛⬛⬛⬜⬜⬜⬛⬛⬛⬜⬛⬜⬛⬛⬛⬛⬜⬛⬛⬛⬛⬛⬛⬛⬛⬛⬛⬛⬛⬛⬛⬛⬛⬛⬜⬜⬜⬜⬜ |
| SB1173 (*n*=2) | 0.2 | C | CB | 2003 | ⬛⬛⬜⬛⬛⬛⬛⬛⬜⬛⬛⬛⬛⬛⬛⬜⬛⬛⬛⬛⬜⬛⬛⬛⬛⬛⬛⬛⬛⬛⬛⬛⬛⬛⬜⬜⬜⬜⬜⬜⬜⬜⬜ |
| SB1174 (*n*=158) | 16.7 | C, RD, WB | CB, PG | 2003, 2004, 2006-2016 | ⬛⬛⬜⬜⬛⬛⬛⬛⬜⬛⬛⬛⬜⬛⬛⬜⬛⬛⬛⬛⬛⬛⬛⬛⬛⬛⬛⬛⬛⬛⬛⬛⬛⬛⬜⬜⬛⬛⬜⬜⬜⬜⬜ |
| SB1190 (*n*=40) | 4.2 | C, RD, WB | CB, PG, BJ | 2007-2016 | ⬛⬜⬜⬛⬛⬛⬛⬛⬜⬛⬛⬛⬛⬛⬛⬜⬛⬛⬛⬛⬜⬛⬛⬛⬛⬛⬛⬛⬛⬛⬛⬛⬛⬛⬛⬛⬜⬛⬜⬜⬜⬜⬜ |
| SB1191 (*n*=2) | 0.2 | RD, WB | CB | 2015, 2016 | ⬛⬛⬜⬛⬛⬛⬛⬛⬜⬛⬛⬜⬜⬜⬛⬜⬛⬛⬛⬛⬜⬛⬛⬛⬛⬛⬛⬛⬛⬛⬛⬛⬛⬛⬛⬛⬛⬛⬜⬜⬜⬜⬜ |
| SB1195 (*n*=42) | 4.4 | C, RD, WB | CB, PG | 2007-2016 | ⬛⬛⬜⬛⬜⬛⬛⬛⬜⬛⬛⬛⬛⬛⬛⬜⬛⬛⬛⬛⬜⬛⬛⬛⬛⬛⬛⬛⬛⬛⬛⬛⬛⬛⬛⬛⬛⬛⬜⬜⬜⬜⬜ |
| SB1230 (*n*=3) | 0.3 | C | PG, BJ | 2006, 2010 | ⬛⬛⬜⬛⬛⬛⬛⬜⬜⬜⬜⬜⬜⬛⬛⬜⬛⬛⬛⬛⬜⬛⬛⬛⬛⬛⬛⬛⬛⬛⬛⬛⬛⬛⬛⬛⬜⬛⬜⬜⬜⬜⬜ |
| SB1232 (*n*=2) | 0.2 | RD, WB | CB, PG | 2010, 2014 | ⬛⬛⬜⬜⬛⬛⬛⬛⬜⬛⬛⬛⬜⬛⬛⬜⬛⬛⬛⬛⬛⬛⬛⬛⬛⬛⬛⬛⬛⬛⬛⬛⬛⬛⬛⬛⬛⬛⬜⬜⬜⬜⬜ |
| SB1254 (*n*=1) | 0.1 | RD | CB | 2010 | ⬛⬛⬜⬛⬛⬛⬛⬛⬜⬛⬛⬛⬛⬛⬛⬜⬛⬛⬛⬛⬜⬛⬛⬛⬛⬛⬛⬜⬛⬛⬛⬛⬛⬛⬛⬛⬛⬛⬜⬜⬜⬜⬜ |
| SB1257 (*n*=2) | 0.2 | WB | CB | 2013, 2015 | ⬛⬛⬜⬛⬛⬛⬛⬛⬜⬛⬜⬛⬛⬛⬛⬜⬛⬛⬛⬛⬜⬛⬛⬛⬛⬛⬛⬛⬛⬛⬛⬛⬛⬛⬛⬛⬛⬛⬜⬜⬜⬜⬜ |
| SB1264 (*n*=92) | 9.7 | C, RD, WB | CB, PG | 2007-2016 | ⬛⬜⬜⬛⬛⬛⬛⬛⬜⬛⬛⬛⬛⬛⬜⬜⬛⬛⬛⬛⬜⬛⬛⬛⬛⬛⬛⬛⬛⬛⬛⬛⬛⬛⬛⬛⬛⬛⬜⬜⬜⬜⬜ |
| SB1265 (*n*=2) | 0.2 | WB | CB | 2012, 2014 | ⬛⬛⬜⬜⬜⬛⬛⬛⬜⬛⬛⬛⬛⬛⬜⬜⬛⬛⬛⬛⬛⬛⬛⬛⬛⬛⬛⬛⬛⬛⬛⬛⬛⬛⬛⬛⬛⬛⬜⬜⬜⬜⬜ |
| SB1266 (*n*=16) | 1.7 | RD, WB | CB | 2007, 2008, 2012, 2015 | ⬜⬛⬜⬛⬛⬛⬛⬛⬜⬛⬛⬛⬛⬛⬜⬜⬛⬛⬛⬛⬜⬛⬛⬛⬛⬛⬛⬛⬛⬛⬛⬛⬛⬛⬛⬛⬛⬛⬜⬜⬜⬜⬜ |
| SB1267 (*n*=1) | 0.1 | RD | CB | 2006 | ⬛⬛⬜⬛⬛⬜⬛⬛⬜⬜⬜⬛⬛⬛⬛⬜⬛⬛⬛⬛⬜⬛⬛⬛⬛⬛⬛⬛⬛⬛⬛⬛⬛⬛⬛⬛⬛⬛⬜⬜⬜⬜⬜ |
| SB1269 (*n*=1) | 0.1 | WB | CB | 2015 | ⬛⬛⬜⬛⬛⬛⬛⬛⬜⬜⬛⬛⬛⬜⬜⬜⬛⬛⬛⬛⬜⬛⬛⬛⬛⬛⬛⬛⬛⬛⬛⬛⬛⬛⬛⬛⬛⬛⬜⬜⬜⬜⬜ |
| SB1273 (*n*=2) | 0.2 | C, RD | PG, BJ | 2007 | ⬛⬛⬜⬛⬛⬛⬛⬜⬜⬜⬛⬛⬛⬜⬜⬜⬛⬛⬛⬛⬜⬛⬛⬛⬛⬛⬛⬛⬛⬛⬛⬛⬛⬛⬛⬛⬛⬛⬜⬜⬜⬜⬜ |
| SB1277 (*n*=1) | 0.1 | C | BJ | 2010 | ⬛⬛⬜⬛⬛⬛⬛⬛⬜⬜⬛⬛⬛⬜⬜⬜⬛⬛⬛⬛⬜⬛⬛⬛⬛⬛⬛⬛⬛⬛⬛⬛⬛⬛⬛⬛⬛⬛⬜⬜⬜⬜⬜ |
| SB1314 (*n*=1) | 0.1 | WB | CB | 2011 | ⬛⬛⬜⬜⬛⬛⬛⬛⬜⬛⬛⬛⬛⬛⬛⬜⬛⬛⬛⬛⬛⬛⬛⬛⬛⬛⬛⬛⬛⬛⬛⬛⬛⬛⬜⬜⬛⬛⬜⬜⬜⬜⬜ |
| SB1333 (*n*=1) | 0.1 | C | PG | 2009 | ⬛⬛⬜⬛⬛⬛⬛⬛⬜⬜⬛⬛⬛⬛⬜⬜⬛⬛⬛⬛⬜⬛⬛⬛⬛⬛⬛⬛⬛⬛⬛⬛⬛⬛⬛⬛⬛⬛⬜⬜⬜⬜⬜ |
| SB1375 (*n*=4) | 0.4 | C, RD | CB | 2010 | ⬛⬜⬜⬛⬜⬜⬛⬛⬜⬛⬛⬛⬛⬛⬛⬜⬛⬛⬛⬛⬛⬛⬛⬛⬛⬛⬛⬛⬛⬛⬛⬛⬜⬛⬛⬛⬛⬛⬜⬜⬜⬜⬜ |
| SB1483 (*n*=12) | 1.3 | C, RD, WB | CB, PG, BJ | 2007-2009, 2012, 2014,2015 | ⬛⬛⬜⬜⬛⬛⬛⬛⬜⬛⬛⬛⬜⬛⬜⬜⬛⬛⬛⬛⬛⬛⬛⬛⬛⬛⬛⬛⬛⬛⬛⬛⬛⬛⬜⬜⬛⬛⬜⬜⬜⬜⬜ |
| SB1484 (*n*=2) | 0.2 | C | CB | 2007, 2009 | ⬛⬛⬜⬛⬛⬛⬛⬛⬜⬛⬛⬛⬛⬛⬜⬜⬛⬛⬛⬛⬜⬜⬛⬛⬛⬛⬛⬛⬛⬛⬛⬛⬛⬛⬛⬛⬛⬛⬜⬜⬜⬜⬜ |
| SB1572 (*n*=1) | 0.1 | WB | BJ | 2013 | ⬛⬛⬜⬛⬛⬛⬛⬛⬜⬛⬛⬛⬛⬛⬛⬜⬛⬛⬛⬛⬛⬛⬛⬛⬛⬛⬛⬛⬛⬛⬛⬛⬛⬛⬛⬛⬛⬜⬜⬜⬜⬜⬜ |
| SB1607 (*n*=5) | 0.5 | C, RD | CB | 2008-2010, 2012 | ⬛⬛⬜⬛⬜⬛⬛⬛⬜⬛⬛⬛⬛⬛⬜⬜⬛⬛⬛⬛⬜⬛⬛⬛⬛⬛⬛⬛⬛⬛⬛⬛⬛⬛⬛⬛⬛⬛⬜⬜⬜⬜⬜ |
| SB1608 (*n*=1) | 0.1 | C | BJ | 2008 | ⬛⬛⬜⬛⬛⬛⬛⬛⬜⬛⬛⬛⬛⬛⬜⬜⬛⬛⬛⬛⬜⬛⬛⬛⬛⬛⬛⬛⬛⬛⬛⬛⬛⬛⬛⬛⬜⬛⬜⬜⬜⬜⬜ |
| SB1609 (*n*=1) | 0.1 | RD | CB | 2008 | ⬛⬛⬜⬛⬜⬛⬛⬛⬜⬛⬛⬛⬛⬛⬜⬜⬛⬛⬛⬛⬜⬛⬛⬛⬛⬛⬛⬛⬛⬛⬜⬛⬛⬛⬛⬛⬛⬛⬜⬜⬜⬜⬜ |
| SB1676 (*n*=1) | 0.1 | RD | CB | 2013 | ⬛⬛⬜⬛⬛⬜⬛⬛⬜⬛⬛⬛⬜⬛⬛⬜⬛⬛⬛⬛⬜⬛⬛⬛⬛⬛⬛⬛⬛⬛⬛⬛⬛⬛⬛⬛⬛⬛⬜⬜⬜⬜⬜ |
| SB1993 (*n*=1) | 0.1 | C | BJ | 2010 | ⬛⬛⬜⬛⬛⬛⬛⬛⬜⬛⬛⬛⬛⬛⬛⬜⬜⬜⬛⬛⬜⬛⬛⬛⬛⬛⬛⬛⬛⬛⬛⬛⬛⬛⬛⬛⬛⬛⬜⬜⬜⬜⬜ |
| SB2354 (*n*=1) | 0.1 | RD | CB | 2015 | ⬛⬛⬜⬛⬜⬛⬛⬛⬜⬛⬛⬛⬛⬛⬛⬜⬛⬛⬛⬛⬜⬛⬜⬛⬛⬛⬛⬛⬛⬛⬛⬛⬛⬛⬛⬛⬛⬛⬜⬜⬜⬜⬜ |
| SB2529 (*n*=1) | 0.1 | RD | CB | 2014 | ⬛⬛⬜⬜⬛⬛⬛⬛⬜⬛⬛⬛⬜⬛⬛⬜⬜⬛⬛⬛⬛⬛⬛⬛⬛⬛⬛⬛⬛⬛⬛⬛⬛⬛⬜⬜⬛⬛⬜⬜⬜⬜⬜ |
| SB2531 (*n*=1) | 0.1 | RD | CB | 2014 | ⬛⬛⬜⬛⬜⬛⬛⬛⬜⬛⬛⬛⬛⬛⬛⬜⬛⬛⬛⬛⬜⬛⬛⬛⬛⬛⬛⬛⬛⬛⬛⬛⬛⬜⬛⬛⬛⬛⬜⬜⬜⬜⬜ |
| SB2530 (*n*=1) | 0.1 | RD | CB | 2016 | ⬜⬛⬜⬛⬛⬛⬛⬛⬜⬛⬛⬜⬛⬛⬛⬜⬛⬛⬛⬛⬜⬛⬛⬛⬛⬛⬛⬛⬛⬛⬛⬛⬛⬛⬛⬛⬛⬛⬜⬜⬜⬜⬜ |

Spoligotyping profile: designation according to M.bovis.org database; (n) indicates the number of isolates.

Host species: C – cattle, RD – red deer and WB – wild boar.

Geographic location: CB – Castelo Branco, PG – Portalegre and BJ – Beja.

**Supplementary Table S2**. Molecular differentiation of *M. bovis* isolates by 8-*loci* MIRU-VNTR.

| **MIRU type** | **MIRU-VNTR profile** | | | | | | | | **Host species** | **Geographic region** | **Year (s) of isolation** |
| --- | --- | --- | --- | --- | --- | --- | --- | --- | --- | --- | --- |
|  | **VNTR3232** | **ETR-A** | **ETR-B** | **ETR-C** | **QUB11a** | **QUB11b** | **MIRU26** | **MIRU4** |  |  |  |
| M03 (*n*=2) | 7 | 6 | 4 | 4 | 10 | 2 | 5 | 3 | C | CB, BJ | 2006 |
| M09 (*n*=1) | 7 | 6 | 4 | 2 | 6 | 2 | 5 | 3 | C | BJ | 2006 |
| M12 (*n*=1) | 6 | 5 | 4 | 4 | 6 | 2 | 5 | 3 | C | BJ | 2007 |
| M13 (*n*=1) | 7 | 6 | 4 | 2 | 11 | 2 | 2 | 3 | C | CB | 2004 |
| M13 and M141 (*n*=1) | 7 | 6 | 4 | 2 | 11 | 2+3 | 2 | 3 | C | BJ | 2006 |
| M14 (*n*=1) | 4 | 5 | 2 | 4 | 8 | 2 | 5 | 3 | RD | CB | 2013 |
| M14 and M134 (*n*=1) | 3+4 | 5 | 2 | 4 | 8 | 2 | 5 | 3 | C | BJ | 2008 |
| M15 (*n*=5) | 4 | 6 | 4 | 4 | 10 | 2 | 5 | 3 | C, RD | CB, BJ | 2007, 2008, 2010, 2014 |
| M15 and M143 (*n*=1) | 4 | 6 | 3+4 | 4 | 10 | 2 | 5 | 3 | C | BJ | 2006 |
| M16 (*n*=1) | 7 | 6 | 4 | 4 | 11 | 2 | 5 | 2 | C | BJ | 2006 |
| M26 (*n*=1) | 7 | 6 | 2 | 4 | 10 | 2 | 6 | 3 | C | BJ | 2006 |
| M30 (*n*=1) | 7 | 6 | 4 | 2 | 6 | 1 | 2 | 3 | C | CB | 2007 |
| M33 (*n*=1) | 7 | 6 | 4 | 2 | >12 | 4 | 2 | 3 | C | BJ | 2006 |
| M34 (*n*=35) | 4 | 6 | 4 | 4 | 11 | 2 | 5 | 3 | C, RD, WB | CB, PG, BJ | 2004, 2006-2016 |
| M34 and M134 (*n*=2) | 3+4 | 6 | 4 | 4 | 11 | 2 | 5 | 3 | RD, WB | CB | 2008, 2015 |
| M36 (*n*=1) | 8 | 4 | 3 | 4 | 11 | 2 | 5 | 2 | C | CB | 2002 |
| M42 (*n*=1) | 7 | 5 | 4 | 4 | 11 | 2 | 3 | 2 | RD | CB | 2006 |
| M74 (*n*=1) | 6 | 5 | 4 | 2 | 11 | 2 | 5 | 3 | WB | CB | 2006 |
| M80 (*n*=1) | 6 | 5 | 4 | 2 | 11 | 3 | 5 | 3 | RD | CB | 2007 |
| M82 (*n*=1) | 6 | 1 | 4 | 2 | 11 | 2 | 5 | 3 | WB | BJ | 2003 |
| M85 (*n*=8) | 6 | 4 | 5 | 4 | 11 | 4 | 5 | 3 | C, WB | CB, PG | 2003, 2004, 2015 |
| M91 (*n*=1) | 5 | 7 | 5 | 5 | 11 | 3 | 6 | 3 | C | CB | 2007 |
| M94 (*n*=4) | 5 | 1 | 4 | 2 | 12 | 2 | 6 | 3 | C | BJ | 2007 |
| M102 (*n*=3) | 5 | 6 | 4 | 4 | 11 | 2 | 5 | 3 | C, WB | CB, BJ | 2009, 2014, 2015 |
| M103 (*n*=1) | 3 | 5 | 4 | 4 | 12 | 2 | 5 | 2 | C | PG | 2007 |
| M108 and M109 (*n*=3) | 3+5 | 6 | 4 | 4 | 12 | 2 | 5 | 3 | C, RD | BJ, CB | 2007,2008 |
| M109 (*n*=3) | 5 | 6 | 4 | 4 | 12 | 2 | 5 | 3 | C, RD | CB, PG, BJ | 2007, 2009 |
| M111 (*n*=1) | 5 | 4 | 5 | 4 | 12 | 3 | 5 | 3 | C | CB | 2007 |
| M112 (*n*=1) | 5 | 1 | 4 | 2 | 12 | 2 | 5 | 3 | C | BJ | 2007 |
| M121 (*n*=1) | 6 | 5 | 4 | 2 | 12 | 2 | 6 | 3 | WB | CB | 2008 |
| M122 (*n*=3) | 5 | 5 | 4 | 2 | 12 | 2 | 6 | 3 | RD | CB | 2008 |
| M124 (*n*=1) | 5 | 5 | 4 | 4 | 12 | 2 | 5 | 2 | RD | CB | 2007 |
| M125 (*n*=1) | 7 | 1 | 4 | 4 | 12 | 2 | 5 | 3 | WB | BJ | 2007 |
| M126 (*n*=4) | 5 | 6 | 3 | 4 | 12 | 3 | 5 | 3 | RD, WB | CB | 2007, 2008 |
| M127 (*n*=2) | 5 | 5 | 4 | 2 | 12 | 2 | 5 | 3 | WB | CB | 2008 |
| M145 (*n*=1) | 12 | 6 | 4 | 4 | 11 | 2 | 5 | 3 | C | PG | 2007 |
| M146 (*n*=1) | 10 | 1 | 4 | 2 | 8 | 2 | 5 | 3 | C | PG | 2014 |
| M147 (*n*=1) | 7 | 5 | 4 | 4 | 11 | 2 | 5 | 3' | RD | CB | 2015 |
| M148 (*n*=1) | 6 | 5 | 5 | 5 | 11 | 4 | 5 | 3 | C | PG | 2004 |
| M149 (*n*=1) | 6 | 5 | 4 | 4 | 11 | 2 | 4 | 3' | RD | CB | 2015 |
| M150 (*n*=2) | 5 | 7 | 5 | 5 | 10 | 3 | 5 | 3 | C, RD | CB | 2010, 2011 |
| M151 (*n*=1) | 5 | 6 | 5 | 2 | 11 | 2 | 5 | 3 | RD | CB | 2012 |
| M152 (*n*=2) | 5 | 6 | 4 | 5 | 11 | 2 | 5 | 3 | C, WB | BJ | 2016 |
| M153 (*n*=1) | 5 | 6 | 4 | 4 | >12 | 2 | 5 | 3' | C | CB | 2009 |
| M154 (*n*=2) | 5 | 6 | 4 | 4 | 11 | 2 | 5 | 3' | C | PG | 2009, 2014 |
| M155 (*n*=2) | 5 | 6 | 4 | 4 | 11 | 2 | 5 | 2 | C, WB | CB, BJ | 2007, 2016 |
| M156 (*n*=1) | 5 | 6 | 4 | 4 | 10 | 2 | 5 | 3 | C | CB | 2012 |
| M157 (*n*=1) | 5 | 6 | 4 | 2 | >12 | 2 | 6 | 3 | RD | CB | 2009 |
| M158 (*n*=1) | 5 | 6 | 4 | 2 | >12 | 2 | 5 | 3 | C | CB | 2009 |
| M159 (*n*=1) | 5 | 6 | 4 | 2 | >12 | 2 | 5 | 2 | C | CB | 2009 |
| M160 (*n*=8) | 5 | 6 | 4 | 2 | >12 | 1 | 2 | 3 | C | CB, PG | 2007 |
| M161 (*n*=1) | 5 | 6 | 4 | 2 | 12 | 1 | 2 | 3 | C | CB | 2007 |
| M162 (*n*=1) | 5 | 6 | 4 | 2 | 11 | 2 | 6 | 3 | RD | CB | 2009 |
| M163 (*n*=1) | 5 | 6 | 4 | 2 | 11 | 2 | 3 | 3 | WB | CB | 2015 |
| M164 (*n*=1) | 5 | 6 | 4 | 2 | 10 | 2 | 2 | 3 | C | PG | 2014 |
| M165 (*n*=1) | 5 | 6 | 3 | 4 | >12 | 3 | 6 | 3 | WB | CB | 2009 |
| M166 (*n*=1) | 5 | 6 | 3 | 4 | >12 | 3 | 5 | 3 | C | PG | 2009 |
| M167 (*n*=1) | 5 | 6 | 3 | 4 | >12 | 3 | 3 | 3' | WB | CB | 2009 |
| M168 (*n*=3) | 5 | 6 | 3 | 4 | 11 | 3 | 5 | 3 | C, RD | CB | 2014, 2015 |
| M169 (*n*=1) | 5 | 5 | 4 | 5 | 11 | 2 | 5 | 3 | C | PG | 2016 |
| M170 (*n*=1) | 5 | 5 | 4 | 5 | 11 | 2 | 5 | 2 | WB | PG | 2016 |
| M171 (*n*=1) | 5 | 5 | 4 | 4 | 11 | 3 | 5 | 2 | WB | PG | 2014 |
| M172 (*n*=2) | 5 | 5 | 4 | 4 | 11 | 2 | 5 | 3 | C | CB, PG | 2007, 2011 |
| M173 (*n*=1) | 5 | 5 | 4 | 4 | 11 | 2 | 3 | 3' | WB | CB | 2015 |
| M174 (*n*=1) | 5 | 5 | 4 | 4 | 11 | 2 | 6 | 2 | WB | CB | 2013 |
| M175 (*n*=3) | 5 | 5 | 4 | 4 | 11 | 2 | 5 | 2 | C, RD, WB | CB, PG | 2013 |
| M176 (*n*=1) | 5 | 5 | 4 | 4 | 11 | 2 | 3 | 2 | RD | CB | 2013 |
| M177 (*n*=1) | 5 | 5 | 4 | 2 | 11 | 2 | 5 | 3 | C | CB | 2014 |
| M178 (*n*=4) | 5 | 4 | 5 | 4 | >12 | 4 | 5 | 3 | C | PG | 2009 |
| M179 (*n*=3) | 5 | 4 | 5 | 4 | 12 | 4 | 5 | 3 | C, RD | CB, PG | 2007, 2008 |
| M180 (*n*=7) | 5 | 4 | 5 | 4 | 11 | 4 | 5 | 3 | C, RD, WB | CB, PG | 2009, 2012, 2014, 2015 |
| M181 (*n*=1) | 5 | 4 | 4 | 4 | >12 | 4 | 5 | 3 | C | PG | 2009 |
| M182 (*n*=2) | 5 | 1 | 4 | 2 | 11 | 2 | 5 | 3 | C, WB | PG, BJ | 2013, 2015 |
| M183 (*n*=1) | 5' | 6 | 4 | 2 | > 12 | 1 | 2 | 3 | C | CB | 2008 |
| M184 (*n*=1) | 4 | 7 | 5 | 5 | 10 | 4 | 5 | 3 | C | CB | 2008 |
| M185 (*n*=1) | 4 | 7 | 5 | 5 | 10 | 3 | 5 | 3 | C | PG | 2013 |
| M186 (*n*=4) | 4 | 7 | 5 | 5 | 10 | 3 | 5 | 3 | C, RD | CB | 2004, 2007, 2009, 2010 |
| M187 (*n*=1) | 4 | 7 | 5 | 2 | 11 | 2 | 5 | 3 | WB | CB | 2010 |
| M188 (*n*=1) | 4 | 7 | 4 | 6 | >12 | 2 | 6 | 3 | C | PG | 2016 |
| M189 (*n*=2) | 4 | 7 | 4 | 5 | 11 | 2 | 5 | 3 | C | PG | 2005, 2008 |
| M190 (*n*=1) | 4 | 7 | 4 | 4 | >12 | 1 | 2 | 3 | C | PG | 2007 |
| M191 (*n*=2) | 4 | 7 | 4 | 4 | 11 | 2 | 5 | 3 | C | PG | 2005, 2008 |
| M192 (*n*=4) | 4 | 7 | 4 | 4 | 11 | 2 | 5 | 2 | C, RD | PG | 2006, 2008, 2013 |
| M193 (*n*=2) | 4 | 7 | 3 | 4 | 11 | 3 | 5 | 3 | RD | CB | 2010 |
| M194 (*n*=1) | 4 | 6 | 5 | 5 | 10 | 3 | 5 | 3 | RD | CB | 2009 |
| M195 (*n*=1) | 4 | 6 | 5 | 4 | 10 | 4 | 5 | 3 | WB | PG | 2013 |
| M196 (*n*=1) | 4 | 6 | 5 | 3 | 11 | 2 | 5 | 3 | C | BJ | 2013 |
| M197 (*n*=2) | 4 | 6 | 5 | 3 | 11 | 1 | 5 | 3 | C | CB | 2007 |
| M197 and M198 (*n*=1) | 4+11 | 6 | 5 | 3 | 11 | 1 | 5 | 3 | C | CB | 2007 |
| M199 (*n*=4) | 4 | 6 | 4 | 5 | 11 | 2 | 5 | 3 | C, RD, WB | CB, PG, BJ | 2016 |
| M200 (*n*=4) | 4 | 6 | 4 | 5 | 11 | 2 | 5 | 2 | C, RD, WB | CB, PG | 2010, 2012, 2013, 2016 |
| M201 (*n*=2) | 4 | 6 | 4 | 4 | 12 | 2 | 5 | 3 | WB | CB | 2006, 2012 |
| M202 (*n*=1) | 4 | 6 | 4 | 4 | 11 | 3 | 5 | 3 | WB | CB | 2008 |
| M203 (*n*=2) | 4 | 6 | 4 | 4 | 11 | 2 | 5 | 3' | C | PG | 2014 |
| M203 and M204 (*n*=1) | 4+11 | 6 | 4 | 4 | 11 | 2 | 5 | 3' | C | PG | 2009 |
| M205 (*n*=13) | 4 | 6 | 4 | 4 | 11 | 2 | 5 | 2 | C, RD | CB, BJ | 2007, 2008, 2010 |
| M205 and M206 (*n*=2) | 3+4 | 6 | 4 | 4 | 11 | 2 | 5 | 2 | C | BJ | 2007 |
| M207 (*n*=1) | 4 | 6 | 4 | 4 | 11 | 2 | 2 | 4 | C | BJ | 2007 |
| M208 (*n*=1) | 4 | 6 | 4 | 4 | 11 | 2 | 3 | 2 | WB | CB | 2010 |
| M209 (*n*=1) | 4 | 6 | 4 | 4 | 11 | 2 | 5 | 0 | C | BJ | 2006 |
| M210 (*n*=1) | 4 | 6 | 4 | 4 | 10 | 2 | 2 | 3 | C | PG | 2014 |
| M211 (*n*=1) | 4 | 6 | 4 | 4 | 9 | 2 | 5 | 3 | C | BJ | 2010 |
| M212 (*n*=1) | 4 | 6 | 4 | 3 | >12 | 1 | 2 | 3 | C | CB | 2010 |
| M213 (*n*=1) | 4 | 6 | 4 | 3 | 11 | 2 | 6 | 3 | WB | CB | 2016 |
| M214 (*n*=6) | 4 | 6 | 4 | 3 | 11 | 2 | 5 | 3 | RD, WB | CB | 2007, 2008, 2015, 2016 |
| M215 (*n*=1) | 4 | 6 | 4 | 2 | >12 | 2 | 2 | 3 | C | CB | 2008 |
| M216 (*n*=2) | 4 | 6 | 4 | 2 | >12 | 1 | 3 | 3 | C, WB | CB | 2009, 2011 |
| M217 (*n*=2) | 4 | 6 | 4 | 2 | >12 | 1 | 2 | 3 | C | CB | 2009 |
| M218 (*n*=2) | 4 | 6 | 4 | 2 | 11 | 3 | 5 | 3 | RD | CB | 2009 |
| M219 (*n*=15) | 4 | 6 | 4 | 2 | 11 | 2 | 5 | 3 | C, RD, WB | CB, PG | 2008-2013 |
| M219 and M220 (*n*=2) | 3'+4 | 6 | 4 | 2 | 11 | 2 | 5 | 3 | WB | CB | 2009 |
| M221 (*n*=1) | 4 | 6 | 4 | 2 | 11 | 2 | 5 | 2 | C | CB | 2008 |
| M222 (*n*=1) | 4 | 6 | 4 | 2 | 10 | 2 | 2 | 3 | C | PG | 2015 |
| M223 (*n*=6) | 4 | 6 | 3 | 5 | 11 | 3 | 5 | 3 | C, RD, WB | CB, BJ | 2016 |
| M224 (*n*=1) | 4 | 6 | 3 | 5 | 11 | 3 | 3 | 3 | WB | CB | 2012 |
| M225 (*n*=35) | 4 | 6 | 3 | 4 | 11 | 3 | 5 | 3 | C, RD, WB | CB, PG | 2004,2007-2015 |
| M225 and M226 (*n*=1) | 3+4 | 6 | 3 | 4 | 11 | 3 | 5 | 3 | RD | CB | 2014 |
| M227 (*n*=1) | 4 | 6 | 3 | 4 | 11 | 3 | 4 | 3 | RD | CB | 2013 |
| M228 (*n*=1) | 4 | 6 | 3 | 4 | 11 | 3 | 3 | 3 | RD | CB | 2008 |
| M229 (*n*=1) | 4 | 6 | 3 | 4 | 11 | 3 | 5 | 2 | RD | CB | 2008 |
| M230 (*n*=1) | 4 | 6 | 3 | 4 | 8 | 3 | 5 | 3 | WB | CB | 2008 |
| M231 (*n*=7) | 4 | 6 | 3 | 4 | 8 | 2 | 5 | 3 | C | BJ | 2006-2009 |
| M232 (*n*=4) | 4 | 6 | 3 | 4 | 7 | 2 | 5 | 3 | C | BJ | 2010 |
| M233 (*n*=2) | 4 | 6 | 3 | 4 | 6 | 2 | 5 | 3 | C | BJ | 2010 |
| M234 (*n*=1) | 4 | 6 | 3 | 4 | 6 | 2 | 2 | 3 | C | BJ | 2013 |
| M235 (*n*=1) | 4 | 6 | 3 | 2 | 11 | 3 | 5 | 3 | RD | CB | 2015 |
| M236 (*n*=2) | 4 | 5 | 5 | 5 | 11 | 4 | 5 | 3 | C, WB | PG | 2009, 2013 |
| M237 (*n*=5) | 4 | 5 | 5 | 4 | 11 | 4 | 5 | 3 | C, RD, WB | CB, PG | 2006, 2008, 2010, 2011, 2013 |
| M238 (*n*=1) | 4 | 5 | 4 | 5 | 11 | 2 | 5 | 3 | C | PG | 2016 |
| M239 (*n*=4) | 4 | 5 | 4 | 5 | 11 | 2 | 5 | 2 | C, RD, WB | CB, PG | 2016 |
| M240 (*n*=2) | 4 | 5 | 4 | 5 | 11 | 2 | 3 | 2 | WB | CB | 2016 |
| M241 (*n*=9) | 4 | 5 | 4 | 4 | 11 | 2 | 5 | 3' | RD, WB | CB, PG | 2009, 2014, 2015 |
| M241 and M242 (*n*=1) | 1+4 | 5 | 4 | 4 | 11 | 2 | 5 | 3' | WB | CB | 2015 |
| M243 (*n*=6) | 4 | 5 | 4 | 4 | 11 | 2 | 5 | 3 | C, RD, WB | CB, PG | 2011-2014 |
| M244 (*n*=3) | 4 | 5 | 4 | 4 | 11 | 2 | 4 | 3' | RD, WB | CB | 2009, 2014, 2015 |
| M245 (*n*=10) | 4 | 5 | 4 | 4 | 11 | 2 | 5 | 2 | C, RD, WB | CB, PG | 2008, 2011-2014 |
| M245 and M246 (*n*=1) | 3+4 | 5 | 4 | 4 | 11 | 2 | 5 | 2 | RD | CB | 2014 |
| M247 (*n*=1) | 4 | 5 | 4 | 4 | 11 | 2 | 4 | 3 | WB | CB | 2012 |
| M248 (*n*=3) | 4 | 5 | 4 | 4 | 11 | 2 | 3 | 2 | RD, WB | CB | 2011, 2012 |
| M249 (*n*=2) | 4 | 5 | 4 | 3 | 11 | 2 | 5 | 3 | RD | CB | 2006, 2016 |
| M250 (*n*=1) | 4 | 5 | 4 | 3 | 11 | 2 | 2 | 3 | RD | CB | 2016 |
| M251 (*n*=14) | 4 | 5 | 4 | 2 | 11 | 2 | 5 | 3 | C, RD, WB | CB | 2008-2011, 2013-2016 |
| M251 and M252 (*n*=1) | 3+4 | 5 | 4 | 2 | 11 | 2 | 5 | 3 | RD | BJ | 2014 |
| M253 (*n*=1) | 4 | 5 | 4 | 2 | 11 | 2 | 2 | 3 | WB | CB | 2015 |
| M254 (*n*=1) | 4 | 5 | 4 | 2 | 11 | 2 | 5 | 2 | WB | CB | 2015 |
| M255 (*n*=2) | 4 | 5 | 3 | 4 | 11 | 3 | 5 | 3 | RD | CB, PG | 2015 |
| M256 (*n*=2) | 4 | 5 | 3 | 4 | 11 | 2 | 5 | 3 | C | PG, BJ | 2010, 2014 |
| M257 (*n*=1) | 4 | 5 | 3 | 2 | 11 | 2 | 5 | 3 | C | PG | 2014 |
| M258 (*n*=2) | 4 | 5 | 2 | 4 | 9 | 2 | 5 | 3 | RD | CB | 2009, 2014 |
| M259 (*n*=1) | 4 | 5 | 1 | 4 | 8 | 2 | 5 | 3 | C | CB | 2014 |
| M260 (*n*=6) | 4 | 4 | 5 | 5 | 11 | 4 | 5 | 3 | C, RD, WB | CB, PG | 2013, 2016 |
| M261 (*n*=37) | 4 | 4 | 5 | 4 | 11 | 4 | 5 | 3 | C, RD, WB | CB, PG | 2006, 2008-2015 |
| M261 and M262 (*n*=1) | 4+11 | 4 | 5 | 4 | 11 | 4 | 5 | 3 | C | PG | 2014 |
| M261 and M263 (*n*=1) | 4 | 4 | 3+5 | 4 | 11 | 4 | 5 | 3 | RD | CB | 2007 |
| M261 and M264 (*n*=2) | 3+4 | 4 | 5 | 4 | 11 | 4 | 5 | 3 | RD, WB | CB | 2009, 2014 |
| M265 (*n*=1) | 4 | 4 | 5 | 4 | 11 | 4 | 3 | 3 | C | PG | 2013 |
| M266 (*n*=2) | 4 | 4 | 4 | 4 | 11 | 4 | 5 | 3 | C, WB | CB | 2012, 2014 |
| M267 (*n*=1) | 4 | 4 | 3 | 4 | 11 | 3 | 5 | 3 | RD | CB | 2011 |
| M268 (*n*=1) | 4 | 3 | 5 | 4 | 11 | 4 | 6 | 3 | WB | CB | 2012 |
| M269 (*n*=1) | 4 | 3 | 5 | 4 | 10 | 4 | 5 | 3 | WB | CB | 2014 |
| M270 (*n*=1) | 4 | 3 | 5 | 4 | 10 | 4 | 4 | 3 | C | BJ | 2015 |
| M271 (*n*=1) | 4 | 3 | 3 | 4 | 11 | 2 | 5 | 3 | WB | CB | 2008 |
| M272 (*n*=1) | 4 | 2 | 5 | 4 | 11 | 3 | 5 | 3 | C | BJ | 2009 |
| M273 (*n*=1) | 4 | 2 | 5 | 4 | 11 | 2 | 5 | 3 | C | BJ | 2010 |
| M274 (*n*=1) | 4 | 2 | 4 | 2 | 11 | 2 | 5 | 3 | RD | CB | 2012 |
| M275 (*n*=1) | 4 | 1 | 4 | 4 | 11 | 2 | 5 | 3 | RD | BJ | 2015 |
| M276 (*n*=6) | 4 | 1 | 4 | 3 | 11 | 2 | 5 | 3 | C, RD, WB | BJ | 2007, 2010, 2012, 2016 |
| M276 and M277 (*n*=1) | 3+4 | 1 | 4 | 3 | 11 | 2 | 5 | 3 | RD | BJ | 2008 |
| M278 (*n*=1) | 4 | 1 | 4 | 3 | 11 | 2 | 2 | 3 | WB | BJ | 2012 |
| M279 (*n*=4) | 4 | 1 | 4 | 2 | 11 | 2 | 5 | 3 | C, RD, WB | BJ | 2010, 2013, 2015 |
| M280 (*n*=1) | 4 | 1 | 4 | 2 | 11 | 2 | 2 | 3 | C | CB | 2004 |
| M281 (*n*=1) | 3' | 5 | 4 | 4 | 11 | 2 | 5 | 2 | RD | CB | 2006 |
| M282 (*n*=1) | 3' | 5 | 4 | 4 | 11 | 2 | 4 | 2 | RD | CB | 2006 |
| M283 (*n*=1) | 3 | 4 | 5 | 4 | 11 | 4 | 5 | 3 | WB | CB | 2015 |
| M284 and M285 (*n*=1) | 5+6 | 6 | 4 | 2 | 11 | 1 | 5 | 3 | RD | CB | 2015 |
| M286 and M287 (*n*=1) | 4+11 | 7 | 4 | 5 | 11 | 2 | 5 | 2 | C | PG | 2008 |
| M288 and M289 (*n*=1) | 4+11 | 5 | 2 | 4 | 10 | 2 | 5 | 3 | C | CB | 2014 |
| M290 and M291 (*n*=1) | 4+7 | 2 | 3 | 4 | 11 | 2 | 5 | 3 | C | PG | 2007 |
| M292 and M293 (*n*=1) | 3'+4 | 4 | 5 | 4 | 11 | 4 | 5 | 4 | RD | PG | 2014 |
| M294 and M295 (*n*=1) | 3'+4 | 6 | 3 | 4 | 11 | 4 | 5 | 3 | RD | CB | 2008 |
| M296 and M297 (*n*=1) | 5 | 6 | 4 | 2 | 11 | 2 | 1+5 | 3 | WB | CB | 2015 |
| M298 and M299 (*n*=1) | 1 | 1 | 4 | 2+4 | 11 | 2 | 5 | 3 | RD | BJ | 2011 |
| *n*=1 | 1 | 4 | 5 | 2+4 | 7 | 2+4 | 1 | 3 | WB | CB | 2010 |
| *n*=1 | 3'+4 | 6 | 4 | 4 | 11 | 2 | 2+3 | 5 | RD | CB | 2007 |
| *n*=1 | 4 | 5+6 | 3+4 | 4 | 11 | 2 | 3 | 5 | RD | CB | 2009 |
| *n*=1 | 4 | 4+6 | 3+5 | 4 | 11 | 3+4 | 3 | 5 | WB | CB | 2014 |
| *n*=1 | 4 | 5+6 | 3+4 | 4 | 11 | 2+3 | 2+3 | 5 | WB | CB | 2015 |
| *n*=1 | 4 | 6 | 3+4 | 2+4 | 11 | 2+3 | 3 | 5 | RD | CB | 2008 |

MIRU type: (*n*) indicates the number of isolates. Allelic designation 3' means the absence of a 53 bp additional sequence.

Host species: C – cattle, RD – red deer and WB – wild boar.

Geographic location: CB – Castelo Branco, PG – Portalegre and BJ – Beja.

**Supplementary Table S3.** Sub-divison of spoligotyping profiles by MIRU type.

| **Spoligotyping profile** | **MIRU type** | **Host species** | **Year(s) of isolation** | **Geographic location** |
| --- | --- | --- | --- | --- |
| SB0119 (*n*=35) | M153 | C | 2009 | CB |
|  | M109 | C | 2009 | CB |
|  | M102 | C | 2009 | BJ |
|  | M154 (*n*=2) | C | 2009, 2014 | PG |
|  | M155 (*n*=2) | C, WB | 2007, 2016 | BJ, CB |
|  | M190 | C | 2007 | PG |
|  | M192 (*n*=3) | C | 2008 | PG |
|  | M197 (*n*=2) | C | 2007 | CB |
|  | M200 (*n*=2) | C, WB | 2013, 2016 | PG, CB |
|  | M202 | WB | 2008 | CB |
|  | M34 (*n*=4) | C, RD, WB | 2007, 2008 | CB |
|  | M203 (*n*=2) | C | 2014 | PG |
|  | M205 (*n*=9) | C | 2007, 2008 | BJ |
|  | M207 | C | 2007 | BJ |
|  | M15 | C | 2008 | BJ |
|  | M241 | RD | 2009 | CB |
|  | M245 | WB | 2008 | CB |
| SB0120 (*n*=8) | M121 | WB | 2008 | CB |
|  | M91 | C | 2007 | CB |
|  | M180 | C | 2012 | PG |
|  | M225 | WB | 2014 | CB |
|  | M239 | WB | 2016 | CB |
|  | M249 | RD | 2006 | CB |
|  | M256 | C | 2010 | BJ |
|  | M273 | C | 2010 | BJ |
| SB0121 (*n*=59) | M145 | C | 2007 | PG |
|  | M36 | C | 2002 | CB |
|  | M16 | C | 2006 | BJ |
|  | M03 (*n*=2) | C | 2006 | BJ, CB |
|  | M33 | C | 2006 | BJ |
|  | M13 | C | 2004 | CB |
|  | M09 | C | 2006 | BJ |
|  | M30 | C | 2007 | CB |
|  | M26 | C | 2006 | BJ |
|  | M42 | RD | 2006 | CB |
|  | M12 | C | 2007 | BJ |
|  | M102 | WB | 2015 | CB |
|  | M156 | C | 2012 | CB |
|  | M164 | C | 2014 | PG |
|  | M169 | C | 2016 | PG |
|  | M172 (*n*=2) | C | 2007, 2011 | CB, PG |
|  | M174 | WB | 2013 | CB |
|  | M187 | WB | 2010 | CB |
|  | M189 | C | 2008 | PG |
|  | M191 | C | 2008 | PG |
|  | M199 (*n*=3) | RD, WB | 2016 | CB, PG |
|  | M34 (*n*=11) | C, RD, WB | 2007, 2008, 2010-2012, 2015 | CB, BJ |
|  | M205 (*n*=3) | C | 2007, 2008 | CB, BJ |
|  | M15 (*n*=4) | C, RD | 2007, 2010, 2014 | CB, BJ |
|  | M210 | C | 2014 | PG |
|  | M211 | C | 2010 | BJ |
|  | M214 | WB | 2008 | CB |
|  | M219 (*n*=2) | C, RD | 2008 | CB |
|  | M222 | C | 2015 | PG |
|  | M225 (*n*=2) | RD, WB | 2008 | CB |
|  | M230 | WB | 2008 | CB |
|  | M238 | C | 2016 | PG |
|  | M243 (*n*=3) | C, WB | 2011, 2014 | PG |
|  | M251 | RD | 2008 | CB |
|  | M14 | RD | 2013 | CB |
|  | M258 | RD | 2009 | CB |
| SB0122 (*n*=46) | M165 | WB | 2009 | CB |
|  | M166 | C | 2009 | PG |
|  | M167 | WB | 2009 | CB |
|  | M168 (*n*=3) | C, RD | 2014, 2015 | CB |
|  | M193 (*n*=2) | RD | 2010 | CB |
|  | M223 (*n*=6) | C, RD, WB | 2016 | CB, BJ |
|  | M224 | WB | 2012 | CB |
|  | M225 (*n*=24) | C, RD, WB | 2004, 2007-2015 | CB |
|  | M227 | RD | 2013 | CB |
|  | M228 | RD | 2008 | CB |
|  | M235 | RD | 2015 | CB |
|  | M255 (*n*=2) | RD | 2015 | CB, PG |
|  | M267 | RD | 2011 | CB |
|  | M276 | WB | 2012 | BJ |
| SB0134 (*n*=2) | M269 | WB | 2014 | CB |
|  | M270 | C | 2015 | BJ |
| SB0140 (*n*=7) | M150 | C | 2011 | CB |
|  | M184 | C | 2008 | CB |
|  | M185 | C | 2013 | PG |
|  | M186 (*n*=3) | C, RD | 2004, 2007, 2010 | CB |
|  | M194 | RD | 2009 | CB |
| SB0265 (*n*=39) | M80 | RD | 2007 | CB |
|  | M74 | WB | 2006 | CB |
|  | M82 | WB | 2003 | CB |
|  | M122 (*n*=2) | RD | 2008 | CB |
|  | M127 | WB | 2008 | CB |
|  | M177 | C | 2014 | CB |
|  | M94 (*n*=4) | C | 2007 | BJ |
|  | M182 (n=2) | C, WB | 2013, 2015 | PG, BJ |
|  | M34 | C | 2009 | PG |
|  | M219 | WB | 2010 | CB |
|  | M249 | RD | 2016 | CB |
|  | M251 (*n*=13) | C, RD, WB | 2009, 2010, 2011, 2013-2016 | CB |
|  | M275 | RD | 2015 | BJ |
|  | M276 (*n*=4) | C, WB | 2007, 2010, 2012, 2016 | BJ |
|  | M278 | WB | 2012 | BJ |
|  | M279 (*n*=4) | C, RD, WB | 2010, 2013, 2015 | BJ |
| SB0295 (*n*=20) | M196 | C | 2013 | BJ |
|  | M199 | C | 2016 | BJ |
|  | M201 (*n*=2) | WB | 2006, 2012 | CB |
|  | M34 (*n*=3) | C, RD, WB | 2006, 2015 | CB, BJ |
|  | M231 (*n*=6) | C | 2006, 2007, 2009 | BJ |
|  | M232 (*n*=4) | C | 2010 | BJ |
|  | M233 (*n*=2) | C | 2010 | BJ |
|  | M234 | C | 2013 | BJ |
| SB1018 (*n*=5) | M122 | RD | 2008 | CB |
|  | M127 | WB | 2008 | CB |
|  | M112 | C | 2007 | BJ |
|  | M274 | RD | 2012 | CB |
|  | M276 | RD | 2012 | BJ |
| SB1090 (*n*=15) | M160 (*n*=8) | C | 2007 | CB, PG |
|  | M161 | C | 2007 | CB |
|  | M183 | C | 2008 | CB |
|  | M212 | C | 2010 | CB |
|  | M215 | C | 2008 | CB |
|  | M216 (*n*=2) | C, WB | 2009, 2011 | CB |
|  | M217 | C | 2009 | CB |
| SB1167 (*n*=4) | M195 | WB | 2013 | PG |
|  | M256 | C | 2014 | PG |
|  | M257 | C | 2014 | PG |
|  | M281 | RD | 2006 | CB |
| SB1172 (*n*=4) | M189 | C | 2005 | PG |
|  | M191 | C | 2005 | PG |
|  | M34 (*n*=2) | C | 2004, 2011 | BJ |
| SB1174 (*n*=72) | M148 | C | 2004 | PG |
|  | M85 (*n*=8) | C, WB | 2003, 2004, 2015 | PG, CB |
|  | M178 (*n*=4) | C | 2009 | PG |
|  | M180 (*n*=6) | C, RD, WB | 2009, 2012, 2014, 2015 | CB, PG |
|  | M181 | C | 2009 | PG |
|  | M225 | C | 2012 | PG |
|  | M236 | WB | 2013 | PG |
|  | M237 (*n*=4) | C, RD | 2006, 2008, 2011, 2013 | CB, PG |
|  | M260 (*n*=6) | C, RD, WB | 2013, 2016 | CB, PG |
|  | M261 (*n*=35) | RD | 2006, 2008-2015 | CB, PG |
|  | M265 | C | 2013 | PG |
|  | M266 (*n*=2) | C, WB | 2012, 2014 | CB |
|  | M268 | WB | 2012 | CB |
|  | M283 | WB | 2015 | CB |
| SB1190 (*n*=17) | M125 | WB | 2007 | BJ |
|  | M152 | C | 2016 | BJ |
|  | M152 | WB | 2016 | BJ |
|  | M102 | C | 2014 | CB |
|  | M34 (*n*=13) | C, RD, WB | 2009-2014 | PG, BJ |
| SB1191 (*n*=2) | M250 | RD | 2016 | CB |
|  | M253 | WB | 2015 | CB |
| SB1195 (*n*=20) | M151 | RD | 2012 | CB |
|  | M157 | RD | 2009 | CB |
|  | M158 | C | 2009 | CB |
|  | M159 | C | 2009 | CB |
|  | M162 | RD | 2009 | CB |
|  | M163 | WB | 2015 | CB |
|  | M213 | WB | 2016 | CB |
|  | M214 (*n*=4) | RD | 2007, 2008, 2015, 2016 | CB |
|  | M218 | RD | 2009 | CB |
|  | M219 (*n*=8) | C, RD, WB | 2008, 2009, 2011-2013 | CB, PG |
| SB1264 (*n*=50) | M147 | RD | 2015 | CB |
|  | M149 | RD | 2015 | CB |
|  | M170 | WB | 2016 | PG |
|  | M124 | RD | 2007 | CB |
|  | M171 | WB | 2014 | PG |
|  | M173 | WB | 2015 | CB |
|  | M175 (*n*=3) | C, RD, WB | 2013 | CB, PG |
|  | M176 | RD | 2013 | CB |
|  | M192 | RD | 2013 | PG |
|  | M200 (*n*=2) | RD, WB | 2010, 2012 | CB, PG |
|  | M205 | RD | 2010 | CB |
|  | M208 | WB | 2010 | CB |
|  | M219 (*n*=2) | RD, WB | 2009 | CB |
|  | M239 (*n*=3) | C, RD | 2016 | CB, PG |
|  | M240 (*n*=2) | WB | 2016 | CB |
|  | M241 (*n*=8) | RD, WB | 2009, 2014, 2015 | CB, PG |
|  | M243 (*n*=3) | C, RD, WB | 2012, 2013 | CB, PG |
|  | M244 (*n*=3) | RD, WB | 2009, 2014, 2015 | CB |
|  | M245 (*n*=9) | C, RD, WB | 2008, 2011-2014 | CB, PG |
|  | M247 | WB | 2012 | CB |
|  | M248 (*n*=3) | RD, WB | 2011, 2012 | CB |
|  | M103 | C | 2007 | PG |
| SB1266 (*n*=12) | M126 (*n*=4) | RD, WB | 2007, 2008 | CB |
| SB1266 | M225 (*n*=7) | RD, WB | 2008, 2012, 2015 | CB |
| SB1266 | M229 | RD | 2008 | CB |
| SB1273 (*n*=2) | M109 (*n*=2) | C, RD | 2007 | PG, BJ |
| SB1375 (*n*=2) | M258 | RD | 2014 | CB |
|  | M259 | C | 2014 | CB |
| SB1483 (*n*=7) | M179 (*n*=3) | C, RD | 2007, 2008 | CB, PG |
|  | M111 | C | 2007 | CB |
|  | M236 | C | 2009 | PG |
|  | M261 (*n*=2) | RD, WB | 2014, 2015 | CB, PG |
| SB1607 (*n*=4) | M214 | RD | 2008 | CB |
|  | M218 | RD | 2009 | CB |
|  | M219 | C | 2010 | CB |
|  | M221 | C | 2008 | CB |

Spoligotyping profile: designation according with M. bovis.org database; (*n*) indicates the number of isolates.
Host species: C – cattle, RD – red deer and WB – wild boar
Geographic location: CB – Castelo Branco, PG – Portalegre and BJ – Beja.

**Supplementary Table S4.** Additional molecular differentiation of double allele MIRU types.

| **MIRU type** | **MIRU-VNTR profile** | | | | | | | | | | | | **Host species** | **Geographic region** | **Year (s) of isolation** |
| --- | --- | --- | --- | --- | --- | --- | --- | --- | --- | --- | --- | --- | --- | --- | --- |
|  | **VNTR**  **3232** | **ETR-A** | **ETR-B** | **ETR-C** | **QUB**  **11a** | **QUB**  **11b** | **MIRU**  **26** | **MIRU**  **4** | **MIRU**  **16** | **MIRU**  **40** | **ETR-E** | **QUB**  **26** |  |  |  |
| M13 and M141 (*n*=1) | 7 | 6 | 4 | 2 | 11 | 2+3 | 2 | 3 | *i* | | | | C | BJ | 2006 |
| M14 and M134 (*n*=1) | 3+4 | 5 | 2 | 4 | 8 | 2 | 5 | 3 | 4 | 2 | 3 | - | C | BJ | 2008 |
| M15 and M143 (*n*=1) | 4 | 6 | 3+4 | 4 | 10 | 2 | 5 | 3 | 3 | 2 | 3 | 5 | C | BJ | 2006 |
| M34 and M134 (*n*=1) | 3+4 | 6 | 4 | 4 | 11 | 2 | 5 | 3 | 4 | 2 | 2 | - | RD | CB | 2008 |
| M34 and M134 (*n*=1) | 3+4 | 6 | 4 | 4 | 11 | 2 | 5 | 3 | - | 2 | 3 | 5 | WB | CB | 2015 |
| M108 and M109 (*n*=2) | 3+5 | 6 | 4 | 4 | 12 | 2 | 5 | 3 | 3 | 2 | 3 | 5 | RD | BJ, CB | 2007.2008 |
| M108 and M109 (*n*=1) | 3+5 | 6 | 4 | 4 | 12 | 2 | 5 | 3 | *i* | | | | C | BJ | 2008 |
| M197 and M198 (*n*=1) | 4+11 | 6 | 5 | 3 | 11 | 1 | 5 | 3 | 4 | 2 | 3 | - | C | CB | 2007 |
| M203 and M204 (*n*=1) | 4+11 | 6 | 4 | 4 | 11 | 2 | 5 | 3' | 3 | 2 | 3 | 4 | C | PG | 2009 |
| M205 and M206 (*n*=2) | 3+4 | 6 | 4 | 4 | 11 | 2 | 5 | 2 | 3 | 2 | 3 | 4 | C | BJ | 2007 |
| M219 and M220 (*n*=1) | 3'+4 | 6 | 4 | 2 | 11 | 2 | 5 | 3 | 4 | 2 | 3 | 4 | WB | CB | 2009 |
| M219 and M220 (*n*=1) | 3'+4 | 6 | 4 | 2 | 11 | 2 | 5 | 3 | - | 2 | 3 | - | WB | CB | 2009 |
| M225 and M226 (*n*=1) | 3+4 | 6 | 3 | 4 | 11 | 3 | 5 | 3 | 4 | 2 | 3 | 4 | RD | CB | 2014 |
| M241 and M242 (*n*=1) | 1+4 | 5 | 4 | 4 | 11 | 2 | 5 | 3' | 3 | 2 | 3 | - | WB | CB | 2015 |
| M245 and M246 (*n*=1) | 3+4 | 5 | 4 | 4 | 11 | 2 | 5 | 2 | 3 | 2 | 3 | 4 | RD | CB | 2014 |
| M251 and M252 (*n*=1) | 3+4 | 5 | 4 | 2 | 11 | 2 | 5 | 3 | - | 2 | 3 | 5 | RD | BJ | 2014 |
| M261 and M262 (*n*=1) | 4+11 | 4 | 5 | 4 | 11 | 4 | 5 | 3 | 4 | 2 | 3 | 4 | C | PG | 2014 |
| M261 and M263 (*n*=1) | 4 | 4 | 3+5 | 4 | 11 | 4 | 5 | 3 | *i* | | | | RD | CB | 2007 |
| M261 and M264 (*n*=2) | 3+4 | 4 | 5 | 4 | 11 | 4 | 5 | 3 | 4 | 2 | 3 | 4 | RD, WB | CB | 2009, 2014 |
| M276 and M277 (*n*=1) | 3+4 | 1 | 4 | 3 | 11 | 2 | 5 | 3 | 4 | 2 | 3 | 3 | RD | BJ | 2008 |
| M284 and M285 (*n*=1) | 5+6 | 6 | 4 | 2 | 11 | 1 | 5 | 3 | 4 | 2 | 5 | 4 | RD | CB | 2015 |
| M286 and M287 (*n*=1) | 4+11 | 7 | 4 | 5 | 11 | 2 | 5 | 2 | 4 | 2 | 3 | 4 | C | PG | 2008 |
| M288 and M289 (*n*=1) | 4+11 | 5 | 2 | 4 | 10 | 2 | 5 | 3 | 4 | 2 | 3 | 4 | C | CB | 2014 |
| M290 and M291 (*n*=1) | 4+7 | 2 | 3 | 4 | 11 | 2 | 5 | 3 | *i* | | | | C | PG | 2007 |
| M292 and M293 (*n*=1) | 3'+4 | 4 | 5 | 4 | 11 | 4 | 5 | 4 | - | 2 | 4 | - | RD | PG | 2014 |
| M294 and M295 (*n*=1) | 3'+4 | 6 | 3 | 4 | 11 | 4 | 5 | 3 | 4 | 2 | 3 | 4 | RD | CB | 2008 |
| M296 and M297 (*n*=1) | 5 | 6 | 4 | 2 | 11 | 2 | 1+5 | 3 | 4 | 2 | 3 | 5 | WB | CB | 2015 |
| M298 and M299 (*n*=1) | 1 | 1 | 4 | 2+4 | 11 | 2 | 5 | 3 | 4 | 2 | 3 | 4 | RD | BJ | 2011 |

MIRU type: (n) indicates the number of isolates; allelic designation 3' means the absence of a 53 bp additional sequence; -, indicates na undetermined allele; i, indicates the absence of DNA to further proceed with differentiation.

Host species: C - cattle, RD - red deer and WB - wild boar.

Geographic region: CB - Castelo Branco, PG - Portalegre and BJ - Beja.

**Supplementary Table S7**. Number of isolates within each *M. bovis* population *per* geographic location and host species in Portugal.

| **Geographic region** | **AM1 (*n*=112)** | **AM2 (*n*=90)** | **AM3 (*n*=106)** | **AM4 (*n*=63)** | **AM5 (*n*=116)** |
| --- | --- | --- | --- | --- | --- |
| Castelo Branco (*n*=276) | 62 | 68 | 44 | 35 | 67 |
| Portalegre (*n*=119) | 31 | 4 | 58 | 15 | 11 |
| Beja (*n*=92) | 20 | 17 | 4 | 13 | 38 |
| **Host species** | **AM1 (*n*=112)** | **AM2 (*n*=90)** | **AM3 (*n*=106)** | **AM4 (*n*=63)** | **AM5 (*n*=116)** |
| Cattle (*n*=211) | 46 | 26 | 43 | 46 | 50 |
| Wild boar (*n*=126) | 32 | 29 | 30 | 10 | 25 |
| Red deer (*n*=150) | 34 | 35 | 33 | 7 | 41 |

**Supplementary table S8.** Data introduced in SPSS for statistical analysis and results.

| **Data introduced in SPSS** | | | | | |
| --- | --- | --- | --- | --- | --- |
|  | AM1 | AM2 | AM3 | AM4 | AM5 |
| Beja | 20 | 17 | 4 | 13 | 38 |
| Castelo Branco | 62 | 68 | 44 | 35 | 67 |
| Portalegre | 30 | 5 | 58 | 15 | 11 |
|  | AM1 | AM2 | AM3 | AM4 | AM5 |
| Cattle | 46 | 26 | 43 | 46 | 50 |
| Wild boar | 32 | 29 | 30 | 10 | 25 |
| Red deer | 34 | 35 | 33 | 7 | 41 |
| **Chi-square test** | | | | | |
|  | Value | df | Asymptotic Significance (2-sided) | |  |
| Pearson Chi-square | 98,616^a^ | 8 | 0,000 | |  |
| Likelihood ratio | 101,538 | 8 | 0,000 | |  |
| N of valid cases | 487 |  |  | |  |

1. 0 cell (,0%) expect count inferior to 5. Minimum expect count is 11,90.


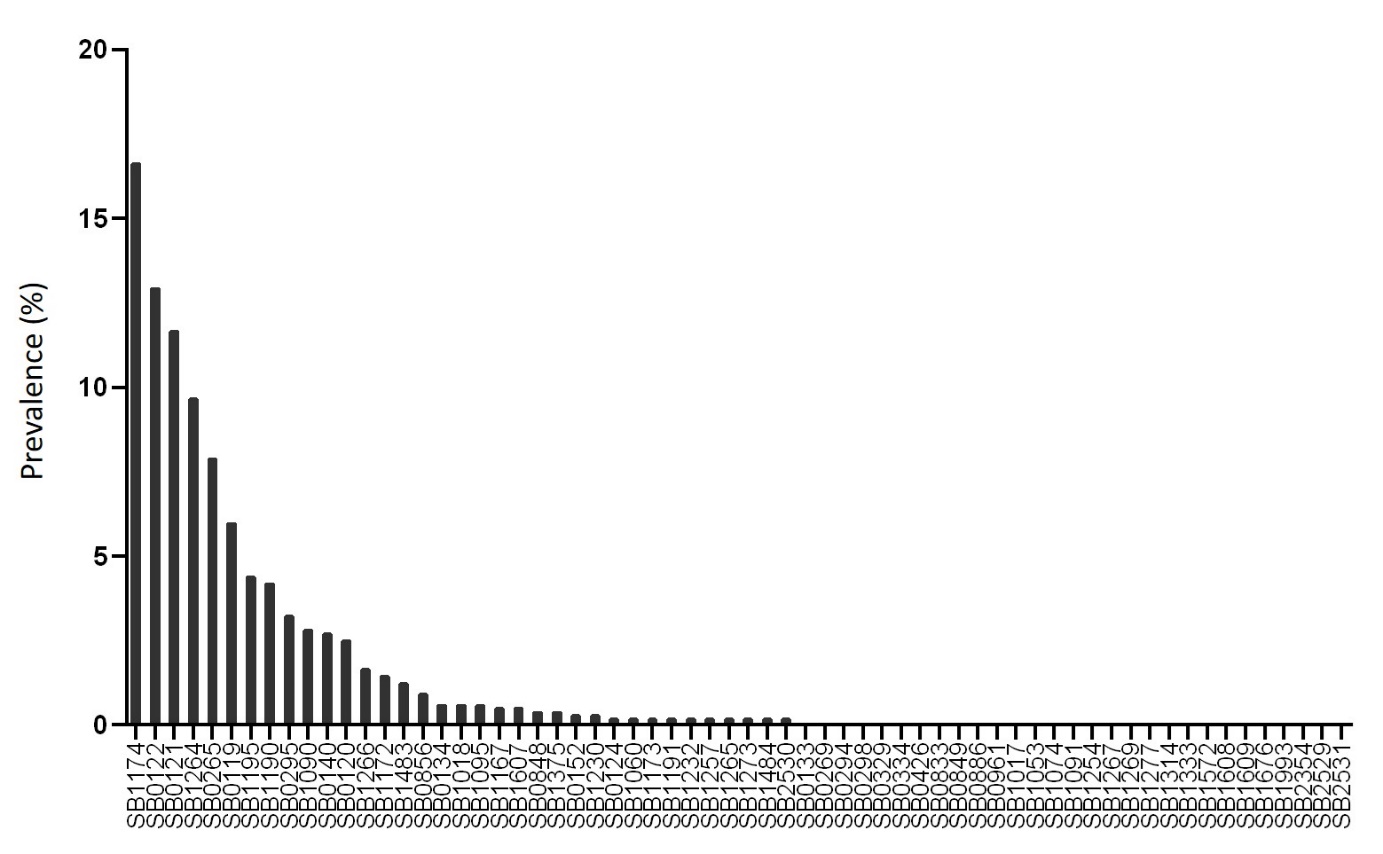


**Supplementary Figure S1.** Distribution of spoligotyping profiles, from higher to lower prevalence (%) in the global dataset.

**
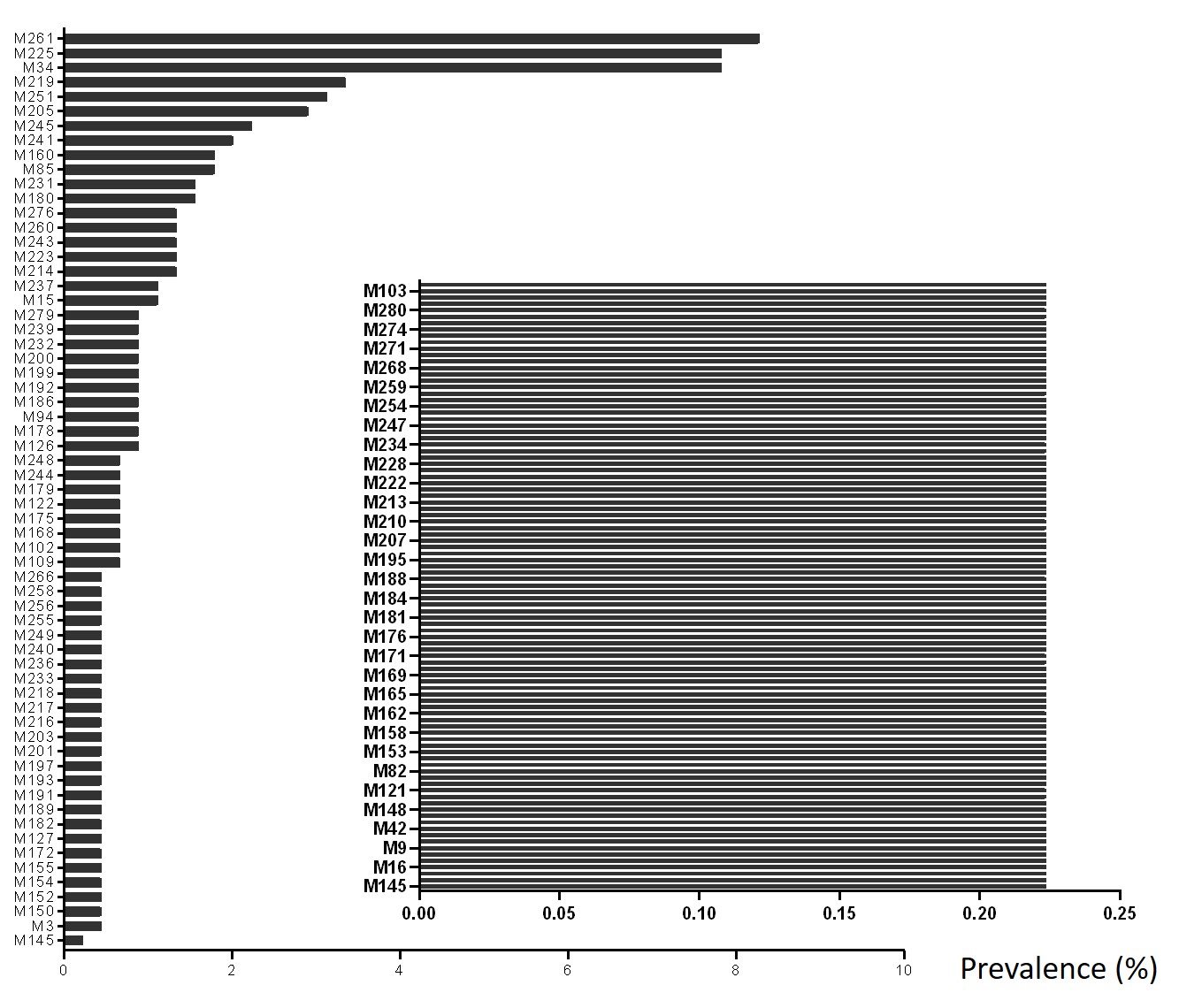
**

**Supplementary Figure S2.** Distribution of MIRU types, from higher to lower prevalence (%) in the global dataset.

**
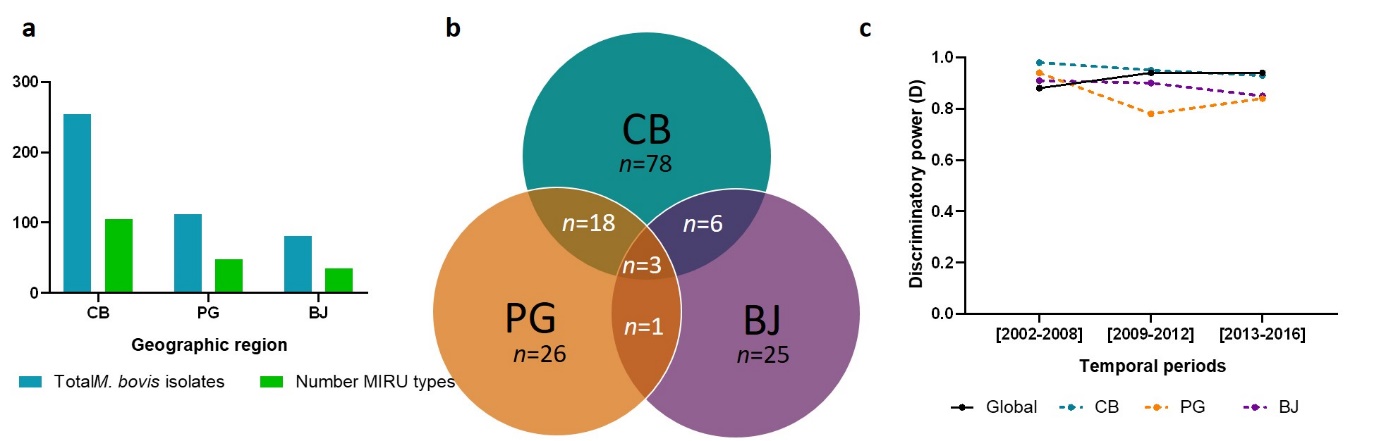
**

**Supplementary Figure S3.** MIRU types analysis per geographic region: (a) comparison between the total number of *M. bovis* isolates and MIRU types registered; (b) common and specific-MIRU types; (c) evolution of the discriminatory power (D values) of MIRU-VNTR, for the global dataset (black line) and for each geographic region. Blue represents Castelo Branco district, orange, the Portalegre district, and, purple, the Beja district.

**
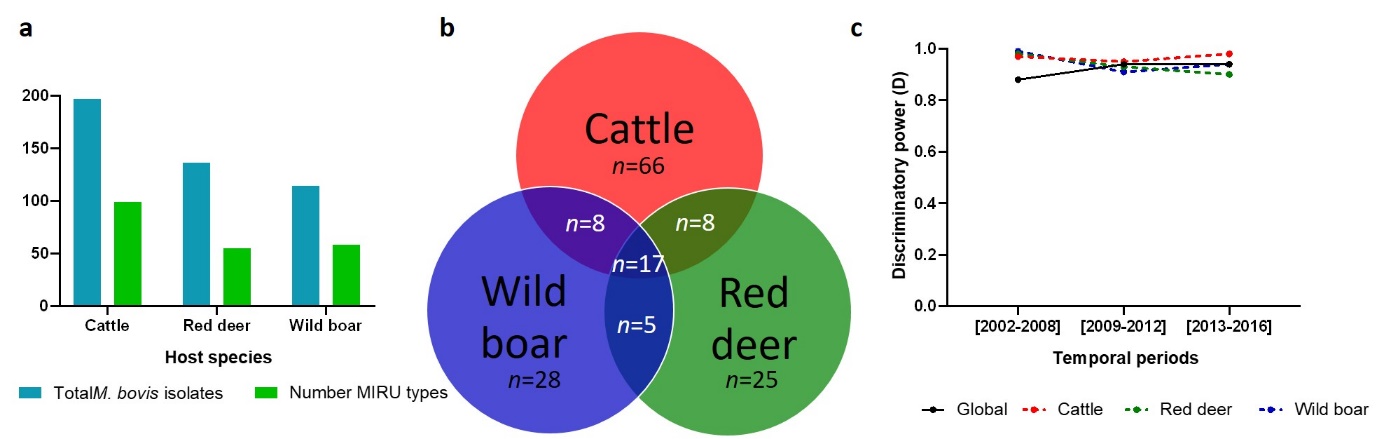
**

**Supplementary Figure S4.** MIRU types analysis per host species: (a) comparison between the total number of *M. bovis* isolates and MIRU types registered; (b) common and specific- MIRU types; (c) evolution of the discriminatory power (D values) of MIRU-VNTR, for the global dataset (black line) and for each host species. Red represents cattle, green for red deer and blue for wild boar.

**Supplementary Figure S5.** Relationship between the number of *M. bovis* isolates recovered from selected livestock herds (LH) and officially-delimited hunting areas (HA) and the corresponding number of spoligotyping profiles obtained.

**
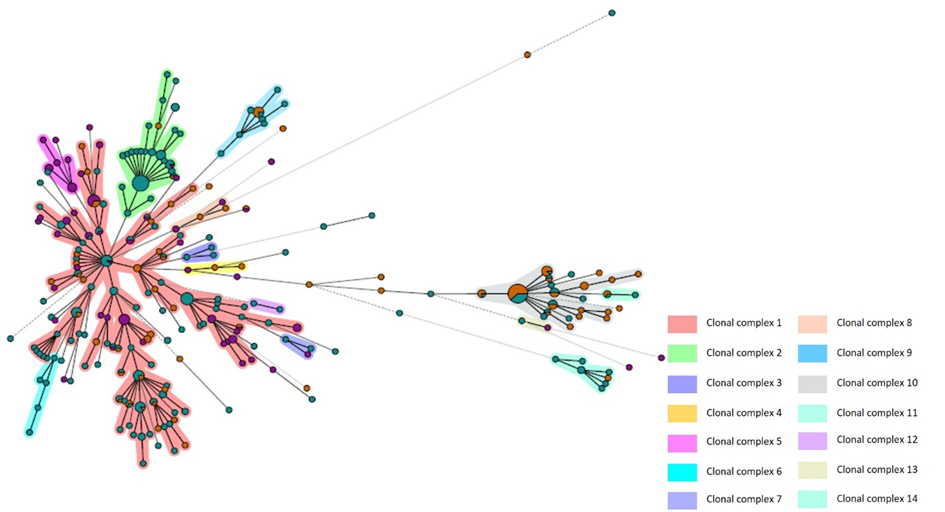
**

**Supplementary Figure S6.** MST analyses illustrating evolutionary relationships among *M. bovis isolates* (n=487) based on combined spoligotyping and 8-loci MIRU-VNTR data, using single locus variant analysis. Circle size is proportional to the number of isolates within each group; nodes are coloured by geographic region (blue for Castelo Branco, orange for Portalegre and purple for Beja); and the different clonal complexes are identified by different colors. The complexity of the lines denotes the number of differences in genotypic (spoligo-MIRU type) profile between two nodes: solid lines (1, 2 or 3 differences), gray dashed lines (4 differences) and gray dotted lines (5 or more differences). Profiles with double alleles were not included in this analysis. MST was generated with Bionumerics (version 6.6, http://www.applied-maths.com/bionumerics).

**
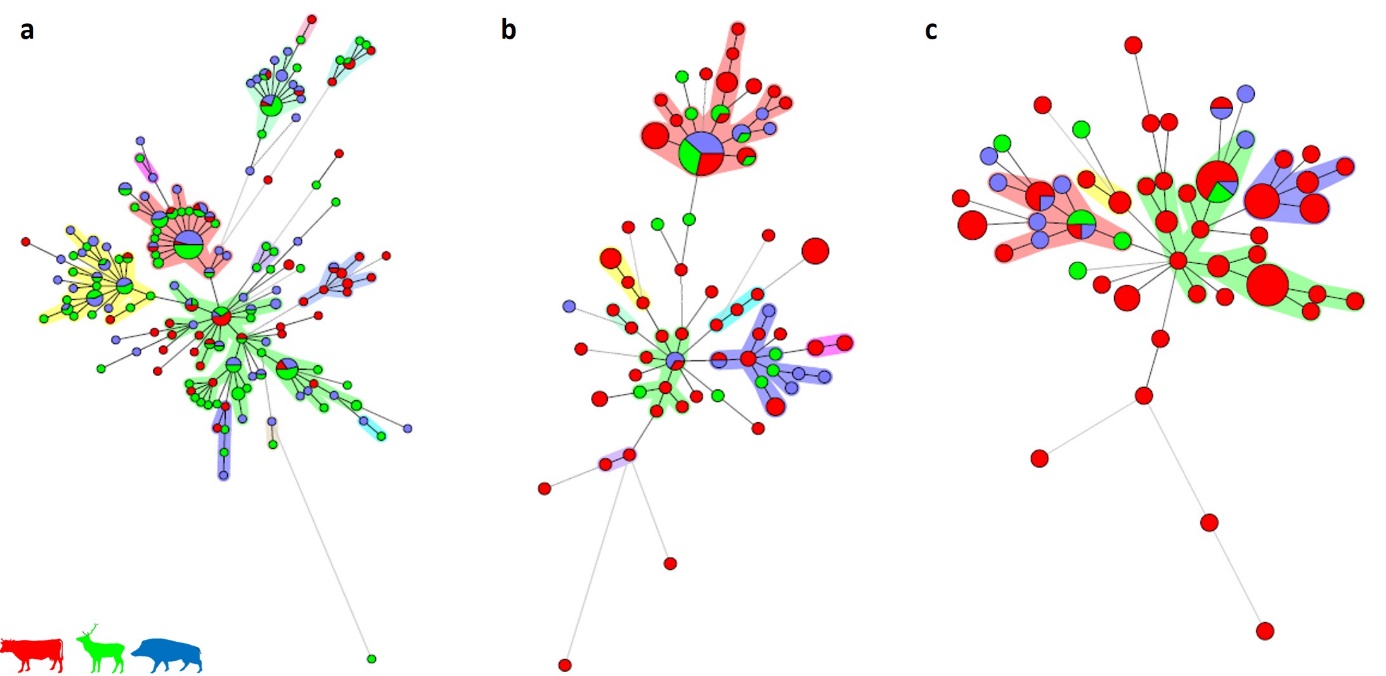
**

**Supplementary Figure S7.** MST analyses illustrating evolutionary relationships among M. bovis isolates in Castelo Branco (a), Portalegre (b) and Beja (c) based on combined spoligotyping and 8-loci MIRU-VNTR data, using single locus variant analysis. Circle size is proportional to the number of isolates within each group; nodes are coloured by host species; and the different clonal complexes are identified by different colors. The complexity of the lines denotes the number of differences in the genotypic (spoligo-MIRU type) profile between two nodes: solid lines (1, 2 or 3 differences), gray dashed lines (4 differences) and gray dotted lines (5 or more differences). Profiles with double alleles were not included in this analysis. MST was generated with Bionumerics (version 6.6, http://www.applied-maths.com/bionumerics).


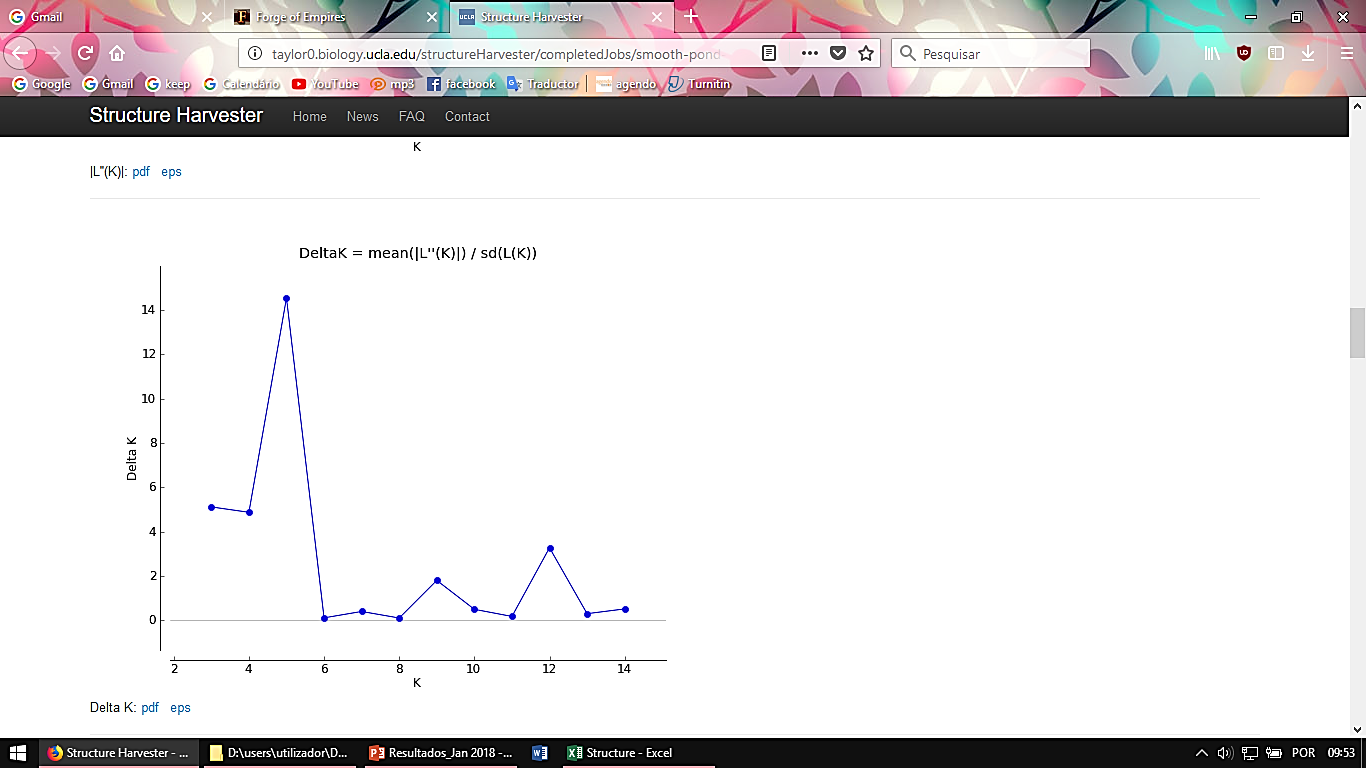


**Supplementary Figure S8.** Selection of K value by estimation by DeltaK = mean(|L''(K)|) / sd(L(K)), using Evanno method.


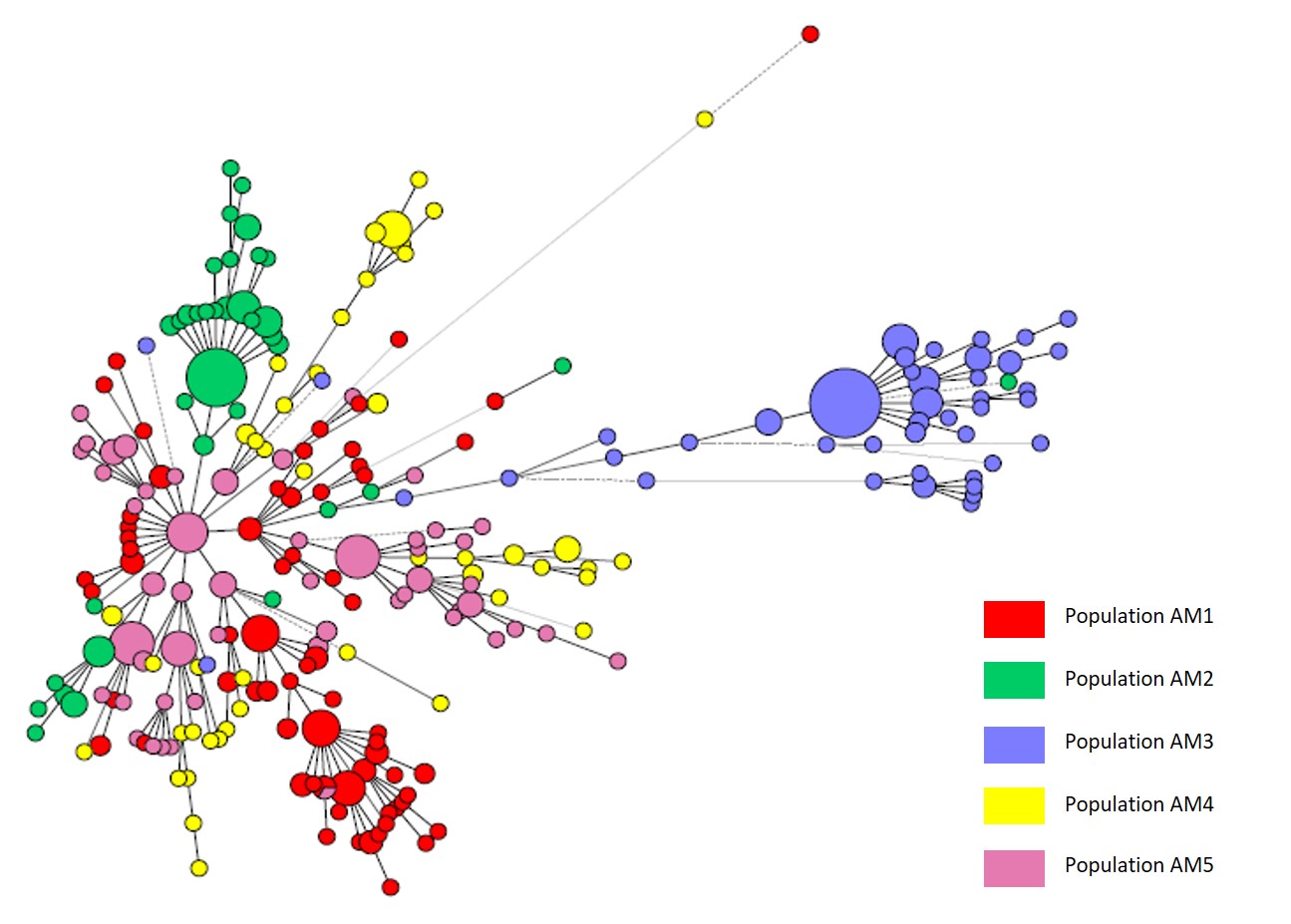


**Supplementary Figure S9.** MST analyses illustrating evolutionary relationships among *M. bovis* isolates (n=487) grouped within ancestral populations AM1 to AM5. Circle size is proportional to the number of isolates within each group; and the complexity of the lines denotes the number of differences in genotypic (spoligo-MIRU type) profile between two nodes: solid lines (1,2 or 3 differences), gray dashed lines (4 differences) and gray dotted lines (5 or more differences). MST was generated with Bionumerics (version 6.6, http://www.applied-maths.com/bionumerics).
